# Zeaxanthin Modulates Early Metabolic and Inflammatory Responses in db/db Mice: Associations with Intestinal Lipid Handling and Gut Microbiome Remodeling

**DOI:** 10.3390/biom16060818

**Published:** 2026-06-01

**Authors:** Yashu Tang, Peiran Lu, Huimin Chen, Siauyen Wong, Md Salahuddin, Mehedi Hasan, Sanmi E. Alake, Yoo Kim, McKale Montgomery, Winyoo Chowanadisai, Brenda J. Smith, Stephen L. Clarke, Edralin A. Lucas, Chwan-Li Shen, Minghua Tang, Dingbo Lin

**Affiliations:** 1 Department of Nutritional Sciences, Oklahoma State University, Stillwater, OK 74078, USA; yastang@okstate.edu (Y.T.); peiran.lu@okstate.edu (P.L.); harmony.chen@okstate.edu (H.C.); siauyen.wong@okstate.edu (S.W.); bau.salahuddin@gmail.com (M.S.); mehedi.hasan10@okstate.edu (M.H.); sanmi.alake@okstate.edu (S.E.A.); yoo.kim@okstate.edu (Y.K.); winyoo.chowanadisai@okstate.edu (W.C.); stephen.clarke@okstate.edu (S.L.C.); edralin.a.lucas@okstate.edu (E.A.L.); 2 Department of Nutritional Sciences, Texas Christian University, Fort Worth, TX 76109, USA; mckale.montgomery@tcu.edu; 3 Department of Obstetrics and Gynecology, Indiana University School of Medicine, Indianapolis, IN 46202, USA; bsm14@iu.edu; 4 Department of Pathology, Texas Tech University Health Sciences Center, Lubbock, TX 79409, USA; leslie.shen@ttuhsc.edu; 5 Department of Food Science and Human Nutrition, Colorado State University, Fort Collins, CO 80523, USA; minghua.tang@colostate.edu

**Keywords:** adiposity, gut microbiome, inflammatory signaling, intestinal lipid transport, type 2 diabetes, zeaxanthin

## Abstract

Dietary zeaxanthin exhibits low intestinal absorption efficiency, and circulating levels are reduced in individuals with type 2 diabetes, suggesting potential metabolic relevance. However, its role during early-stage diabetes remains incompletely understood. This study examined whether dietary zeaxanthin modulates early metabolic and inflammatory responses and influences host–microbiome interactions during early T2DM progression. Four-week-old male db/db mice and wild-type C57BL/6J mice were fed an AIN-93M diet with or without 0.02% (*w*/*w*) zeaxanthin for 4 weeks. Zeaxanthin attenuated body weight gain, adiposity, hyperinsulinemia, and circulating keratinocyte-derived chemokine levels in diabetic mice. These effects were accompanied by reduced ileal membrane localization of Niemann-Pick C1-like protein 1 and decreased hepatic expression of CD36, nuclear factor kappa B p65, and phosphoenolpyruvate carboxykinase 1, without significant improvement in fasting blood glucose or hepatic triglyceride accumulation. Cecal microbiota analysis showed reduced microbial richness in diabetic mice that was not restored by zeaxanthin; however, zeaxanthin induced selective compositional shifts, including enrichment of fermentation-associated taxa (e.g., Ruminococcaceae) and normalization of Clostridium XIVb. Predicted microbial pathways related to fermentation, amino acid biosynthesis, and cofactor metabolism were also altered. Collectively, dietary zeaxanthin modulated early metabolic and inflammatory adaptation and was associated with alterations in intestinal lipid handling, inflammatory signaling, and gut microbiome composition during early T2DM progression.

## 1. Introduction

Type 2 diabetes mellitus (T2DM) is a progressive metabolic disorder characterized by chronic hyperglycemia, insulin resistance, and gradual pancreatic β-cell dysfunction [1,2]. Its global prevalence continues to rise and is projected to exceed 700 million cases by 2045, imposing substantial clinical and socioeconomic burdens [3]. Although pharmacological therapies improve glycemic control, they do not fully prevent disease progression or long-term tissue injury [4]. Consequently, increasing emphasis has been placed on preventive and early-stage interventions, including dietary strategies that enhance metabolic resilience, attenuate the host inflammatory responses, and delay β-cell failure [5,6].

Dietary patterns rich in fruits, vegetables, and bioactive phytochemicals are consistently associated with reduced risk of T2DM and its complications [7,8,9]. Among these compounds, carotenoids and the fruits and vegetables rich in carotenoids have attracted growing interest due to their antioxidant, anti-inflammatory, and immune-modulating properties [10,11,12].

Carotenoids are hydrophobic pigments best known for their roles in vitamin A metabolism, macular pigment maintenance, and visual function, but they also accumulate in metabolically active tissues, including the liver and adipose tissue [13,14]. Dietary carotenoid bioavailability is tightly regulated by intestinal lipid transport mechanisms, including Niemann-Pick C1-like 1 (NPC1L1), scavenger receptor class B type 1 (SCARB1), and fatty acid transporters, such as cluster of differentiation 36 (CD36), in the small intestine [15,16,17,18,19]. Zeaxanthin, an oxygenated carotenoid, has inherently low absorption efficiency, making its systemic availability highly sensitive to changes in intestinal transport and handling [16].

Circulating carotenoid concentrations, including zeaxanthin, are often reduced in humans with obesity, metabolic syndrome, and/or T2DM, likely due to lower dietary intake, altered lipoprotein transport, and increased oxidative turnover [20,21,22,23]. Emerging evidence also suggests that the gut microbiome may influence carotenoid stability, metabolism, and host utilization, implicating carotenoid–microbiota interactions in metabolic regulation [10,24,25]. However, despite these associations, the mechanistic role of zeaxanthin in T2DM remains poorly understood, particularly whether zeaxanthin directly modulates early metabolic dysfunction, intestinal homeostasis, and host–microbiome interactions in mammals.

Rodent models provide a valuable framework for dissecting carotenoid metabolism and function in metabolic disease [26,27]. Leptin receptor-deficient (homozygous LepR^−/−^, or db/db) mice develop early-onset obesity, hyperglycemia, dyslipidemia, and progressive β-cell dysfunction, and are therefore widely used as a genetic model of early-stage T2DM for evaluating preventive nutritional interventions [28,29].

In this study, we sought to investigate the metabolic effects of dietary zeaxanthin in young db/db mice during early T2DM progression. We examined whether zeaxanthin supplementation attenuates early metabolic dysfunction and evaluated associated changes in intestinal lipid transport, inflammatory signaling, hepatic metabolism, and gut microbiome composition and predicted function. We hypothesized that zeaxanthin enhances metabolic resilience by modulating intestinal lipid handling, attenuating inflammation, and reshaping microbiome functional capacity. By integrating metabolic, molecular, and microbial analyses, this study provides insight into the systemic metabolic effects of zeaxanthin and supports its potential as an early nutritional intervention for T2DM.

## 2. Methods

### 2.1. Animals and Diets

Four-week-old male db/db mice and their littermates C57BL/6J wild-type (WT) were generated in-house from heterozygous breeders (LepR^+/−^), that were originally obtained from The Jackson Laboratory (Bar Harbor, ME, USA, Strain #000697). Pups were co-housed with littermates from birth through weaning (3 weeks of age) to minimize variability in maternal, environmental, and early microbial exposures. At weaning, mice were group-housed by sex but not genotype prior to dietary intervention at 4 weeks of age. Animal genotyping was performed by Transnetyx Inc. using automated real-time PCR to confirm leptin receptor genotype (LepR^+/+^(WT), LepR^+/−^, and LepR^−/−^(db/db)) for colony management and experimental assignment. Because the animals were generated from a single inbred breeding colony, the final group sizes (n = 6 per group) were determined by genotype yield and informed by our previous studies [10,12,24,25]. Although a formal power analysis was not performed, the sample sizes were sufficient for statistical analyses.

Male mice were randomly allocated into four dietary treatment groups: WT mice fed an AIN-93M control diet (Control, or C; Research Diets Inc., New Brunswick, NJ, USA, D11112225; Table 1), WT mice fed the Control diet supplemented with 0.02% (*w*/*w*) zeaxanthin (Z or Zeax diet, Research Diets Inc., D18112003; Table 1) [21], db/db mice fed the Control diet, and db/db mice fed Zeax. Zeaxanthin was purchased from Sigma-Aldrich (Catalog #1733122, St. Louis, MO, USA). All diets were vacuum-sealed, protected from light, stored at 4 °C, and replaced every three days to maintain zeaxanthin stability and nutrient integrity. The dietary intervention lasted four weeks, during which food and water were provided ad libitum. Body weight was recorded weekly.

Experimental animals were group-housed (two per cage) to maximize the number of independent cage replicates. Because gut microbiome composition is influenced by coprophagy and shared environment [30], the cage represents the true biological unit; thus, increasing cage number strengthened statistical rigor and reduced the risk of pseudo-replication in microbial analyses.

Animals were housed in the Oklahoma State University (OSU) Laboratory Animal Research Facility, maintained on a 12 h light/12 h dark cycle with controlled temperature and humidity. Bedding was replaced every three days to maintain hygiene and reduce odor and stress that could influence metabolic outcomes. All procedures were approved by the OSU Institutional Animal Care and Use Committee (IACUC protocols #HS-22-55 (breeding, approved date: 9 August 2025)) and #HS-20-75 (research, approval date: 14 November 2023) and were conducted in accordance with NIH guidelines for the Care and Use of Laboratory Animals. Mice were monitored daily, and all efforts were made to minimize discomfort and distress through gentle handling, stable housing conditions, and routine welfare checks. No procedures required anesthesia or analgesia during the study period, and no animals met humane-endpoint criteria for early removal.

### 2.2. Sample Collection and Assessment of Clinical Parameters

Fasting blood glucose (FBG) was measured prior to the initiation of dietary intervention (Baseline) and again following the 4-week dietary treatment (After treatment), immediately before necropsy [31,32]. Mice were fasted for 3 h before glucose assessment, and FBG was determined from tail blood using the Precision Xtra blood glucose monitoring system (Alameda, CA, USA). Following glucose measurement, mice were euthanized under ketamine/xylazine anesthesia (1.25 and 0.625 mL/kg BW, respectively), followed by secondary confirmation of death in accordance with AVMA guidelines.

Blood was collected immediately from the carotid artery, allowed to clot, and centrifuged to obtain serum. Ileum and cecal contents were harvested as previously described for isolation of ileal epithelial/lamina propria tissues and cecal microbiota, and/or fixation for immunohistochemistry [10,11,12]. Epididymal white adipose tissue (eWAT) were rapidly excised, weighed, snap-frozen in liquid nitrogen, and stored at −80 °C for future analyses.

Serum insulin, leptin, ghrelin, and keratinocyte-derived chemokine (KC/CXCL1) were quantified from serum using multiplex bead-based assays, including a customized Bio-Plex Pro^TM^ panel based on the Mouse Cytokine 23-plex Panel (#M60009RDPD) and the Bio-Plex Pro^TM^ Mouse Diabetes 8-plex Panel (#171F7010M) (Bio-Rad Laboratories, Hercules, CA, USA), following the manufacturer’s instructions.

Hepatic triglycerides (TG) were measured according to the manufacturer’s protocol using equal amounts of liver tissue (Cayman Chemical, Ann Arbor, MI, USA, #10010303). Values are expressed as triglyceride concentration in the final sample extracts and were further normalized to tissue weight (µg/mg liver).

### 2.3. Quantitative Real-Time PCR

Total RNA was extracted from frozen liver using TRIzol reagent (Sigma-Aldrich, St. Louis, MO, USA, #T9424), and first-strand cDNA was synthesized using SuperScript II reverse transcriptase (Invitrogen, ThermoFisher Scientific, Waltham, MA, USA, #18064014) following the manufacturer’s protocol as previously described [31]. Quantitative real-time PCR (qPCR) was performed on a Bio-Rad CFX Opus 384 real-time detection system using SYBR Green PCR Master Mix (Thermo Fisher Scientific). Primer sequences are listed in Table 2. Gene expression levels were normalized to glyceraldehyde-3-phosphate dehydrogenase (*GAPDH*), which served as the internal reference gene, and relative expression was calculated using the ΔΔCt method as previously described [31].

### 2.4. Immunoblotting

Total protein was extracted from frozen ileal epithelial/lamina propria or liver tissues using radioimmunoprecipitation assay (RIPA) buffer supplemented with Halt^TM^ protease and phosphatase inhibitor cocktail (ThermoFisher Scientific, Waltham, MA, USA; #78440). Immunoblotting was conducted as previously described [31,33]. Equal amounts of protein (20 μg) were separated by SDS–PAGE, transferred to polyvinylidene difluoride membranes, blocked, and incubated overnight at 4 °C with primary antibodies. Antibodies obtained from Cell Signaling Technology (Danvers, MA, USA) included nuclear factor-κB p65 (NF-κB p65, #8242), phosphorylated dynamin-related protein 1 (p-DRP1, Ser616, #3455), dynamin-related protein 1 (DRP1, #8570), mitofusin-2 (MFN2, #9482), optic atrophy protein-1 (OPA1, #67589), phosphoenolpyruvate carboxykinase-1 (PCK1, #12940), phosphoenolpyruvate carboxykinase-2 (PCK2, #6924), and GAPDH (#5174). Antibodies sourced from Santa Cruz Biotechnology (Dallas, TX, USA) included sterol regulatory element-binding protein-1c (SREBP-1c, #sc-366) and NPC1L1 (#sc-271906). The Proteintech Group (Rosemont, IL, USA) supplied pyruvate dehydrogenase linase-4 (PDK4, #12949-1-AP), overlapping with the m-AAA protease 1 homolog, or mitochondrial metalloendopeptidase (OMA1, #17116-1-AP). Additional antibodies included dynamin-2 (DNM2, Invitrogen/Thermo Fisher Scientific, Carlsbad, CA, USA; #PA5-29017) and β-actin (Invitrogen/Thermo Fisher Scientific; #MA1-140). Protein bands were visualized using the ChemiDoc^TM^ MP Imaging System (Bio-Rad, Hercules, CA, USA) and quantified by densitometry using ImageJ software (Version 1.44, NIH, Bethesda, MD, USA). All protein abundance values were normalized to β-actin or GAPDH and expressed as relative fold change.

### 2.5. Immunohistochemistry for Ileal NPC1L1 and Aminopeptidase N (APN)

Small segments (~2 cm in length) of distal ileum were excised, gently flushed with ice-cold PBS, and fixed in 10% neutral-buffered formalin for 24 h at room temperature. Tissues were then dehydrated through graded ethanol, cleared in xylene, and embedded in paraffin. Sections (5 μm thick) were cut, mounted on charged glass slides, deparaffinized in xylene, and rehydrated through graded alcohols to water. Antigen retrieval was performed in citrate buffer (10 mM, pH 6.0) at 95–98 °C for 15–20 min, followed by cooling to room temperature. Sections were permeabilized in 0.1% Triton X-100 in PBS and blocked for 1 h with 5% normal goat serum in phosphate-buffered saline (PBS).

For immunohistochemistry assessment, sections were incubated overnight at 4 °C with rabbit polyclonal anti-NPC1L1 (Invitrogen, ThermoFisher Scientific, Waltham, MA, USA; #PA1-16800) and rat monoclonal anti-aminopeptidase N/CD13 (APN) (Abcam, Cambridge, UK; #ab33489, clone R3-63). After washing in PBS, sections were incubated for 1 h at room temperature with species-specific secondary antibodies (goat anti-rabbit IgG–Alexa Fluor 488 (#A11034) and goat anti-rat IgG–Alexa Fluor 594 (#R37117), 1:500 dilution; Invitrogen, ThermoFisher Scientific, Waltham, MA, USA). Slides were mounted with antifade medium and imaged using a fluorescence microscope (40× objective). Brush border membrane localization of NPC1L1 and its co-distribution with APN were evaluated using a Keyence All-in-One fluorescence imaging system. For each tissue sample, at least 10 villus regions (fields) were imaged, with a minimum of three high-resolution images captured per field to ensure representative sampling and quantitative reliability [34].

### 2.6. Cecal Microbial DNA Extraction and 16s rRNA Gene Sequencing

Cecal microbial genomic DNA was extracted from cecal contents using the QIAamp PowerFecal Pro DNA Kit (Qiagen, Germantown, MD, USA; #51804) according to the manufacturer’s instructions. The V3–V4 hypervariable region of the bacterial 16S rRNA gene was amplified using Illumina adapter-linked universal primers and sequenced on the Illumina MiSeq platform using 2 × 250 bp paired-end chemistry, with a sequencing depth exceeding 30,000 reads per sample. Raw sequencing data were processed using the Mothur MiSeq Standard Operating Procedure (SOP), including paired-end assembly, quality filtering, alignment to the SILVA v138 reference database, and chimera removal. High-quality sequences were clustered into operational taxonomic units (OTUs) at 97% sequence similarity, and taxonomic classification was performed using the SILVA reference database as previously described [10].

### 2.7. Statistical Analysis and Microbiome Sequencing Bioinformatics

Data were analyzed using GraphPad Prism version 10.1.0 (GraphPad Software, San Diego, CA, USA). Group differences were evaluated by one-way or two-way ANOVA, followed by Tukey’s multiple comparison post hoc test. Pairwise comparisons between two groups were analyzed using an unpaired, two-tailed Student’s *t*-test. Data are expressed as means ± standard error of the mean (SEM), and *p* < 0.05 was considered statistically significant.

For cecal microbiome profiling, microbial alpha diversity was estimated using observed richness (Sobs) and inverse Simpson index, whereas beta diversity was calculated using Bray–Curtis dissimilarity [10]. Principal coordinate analysis (PCoA) was generated in R 4.3.1 (phyloseq + vegan packages) to visualize community structure. Differentially abundant taxa were identified using linear discriminant analysis effect size (LEfSe) with a discrimination threshold of LDA ≥ 3.0. Functional pathway prediction was conducted using PICRUSt2, and differential pathway enrichment was evaluated with ggpicrust2, applying FDR-corrected *p* values < 0.05 as the significance cutoff [35].

## 3. Results

### 3.1. Zeaxanthin Intervention Improves Metabolic Phenotype in db/db Mice

**Figure 1:**
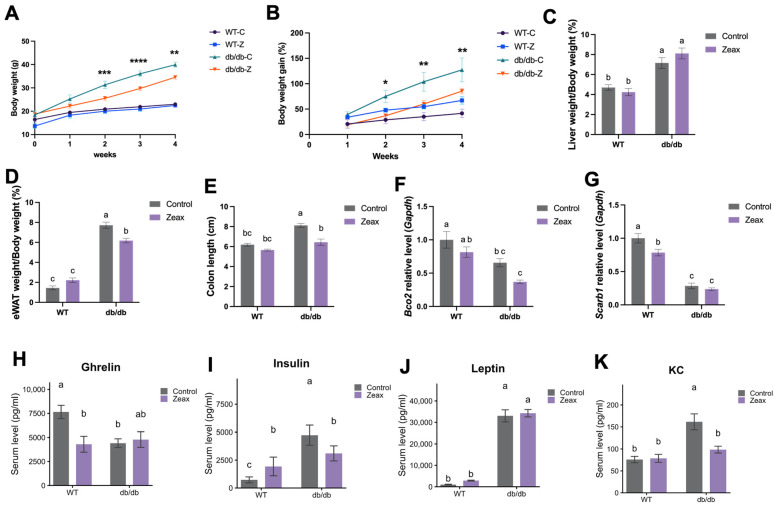
Zeaxanthin improves metabolic phenotype in db/db mice: (**A**) body weight (g) change and (**B**) body weight gain (%) over 4 weeks; (**C**) relative liver weight (liver weight/body weight); (**D**) relative epididymal white adipose tissue (eWAT) weight (eWAT weight/body weight); (**E**) colon length; (**F**,**G**) relative mRNA expression of *Bco2* (**F**) and *Scarb1* (**G**) normalized to *Gapdh*; and (**H**–**K**) serum concentrations of ghrelin (**H**), insulin (**I**), leptin (**J**), and keratinocyte-derived chemokine (KC) (**K**) in male wild-type (WT) and db/db mice fed a control or zeaxanthin-supplemented diet for 4 weeks. Values are presented as mean ± SEM (n = 6 per group). Statistical significance was determined by two-way ANOVA. Means without a common letter differ (*p* < 0.05) (**C**–**K**). * *p* ≤ 0.05, ** *p* ≤ 0.01, *** *p* ≤ 0.001 and **** *p* ≤ 0.0001 indicate differences between db/db-C and db/db-Z in body weight (g) and body weight gain (%). WT, wild type; WT-C, WT mice fed control diet; WT-Z, WT mice fed zeaxanthin diet; db/db-C, db/db mice fed control diet; db/db-Z, db/db mice fed zeaxanthin diet.

After four weeks of dietary zeaxanthin supplementation, db/db mice gained significantly more body weight than WT mice over time. Zeaxanthin supplementation attenuated body weight gain in db/db mice compared with control diet-fed counterparts, whereas no significant effect was observed in WT mice (Figure 1A,B). Zeaxanthin had no effect on liver weight but selectively reduced eWAT mass in db/db mice (Figure 1C,D). Colon length was shorter in zeaxanthin-treated db/db mice relative to their control counterparts (Figure 1E), with no effect detected in WT mice.

To further characterize the animals’ responses to dietary treatment, we examined intestinal lipid-handling pathways. Quantification of ileal β-carotene oxygenase 2 (*Bco2*) and *Scarb1* transcript levels showed that db/db mice exhibited lower expression of both genes compared with WT mice (Figure 1F,G). Zeaxanthin supplementation did not alter *Bco2* or *Scarb1* expression in db/db mice but significantly reduced *Scarb1* expression in WT mice (Figure 1G). Serum metabolic and inflammatory hormones were also assessed (Figure 1H–K). Consistent with early-stage diabetes, db/db mice exhibited markedly reduced ghrelin levels and elevated insulin, leptin, and KC concentrations compared with WT controls. Zeaxanthin supplementation reduced insulin and KC levels in db/db mice, whereas leptin and ghrelin levels remained unchanged. In WT mice, zeaxanthin supplementation decreased ghrelin and increased insulin levels; however, the physiological significance of the increase in serum insulin concentration remains unclear and warrants further investigation.

### 3.2. Zeaxanthin Modulates Brush Border Membrane Localization of NPC1L1

**Figure 2:**
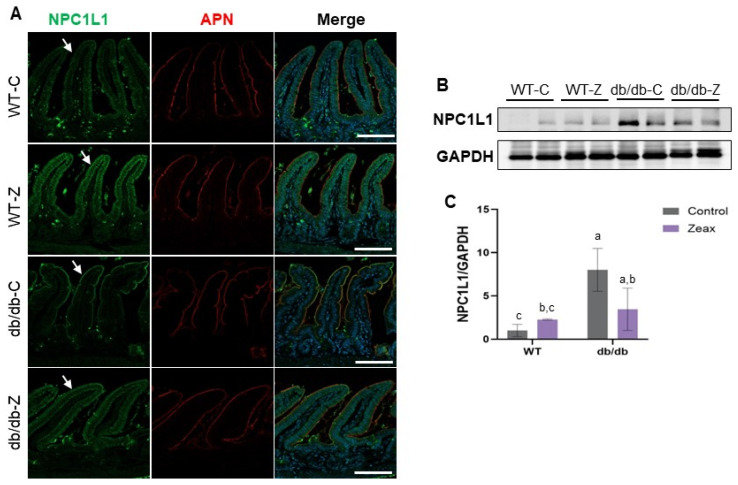
Zeaxanthin modulates brush border membrane localization of Niemann-Pick C1-like 1 (NPC1L1). (**A**) Immunofluorescence staining of NPC1L1 in ileal sections from wild-type (WT) and db/db mice fed a control or zeaxanthin-supplemented diet. Images are shown at 20× magnification; scale bar, 50 μm. NPC1L1 is shown in green, and aminopeptidase N (APN) in red. (**B**) Representative immunoblot of NPC1L1 protein in ileal tissue lysates with quantification shown in (**C**). Values are presented as mean ± SEM (n = 6/group). Means without a common letter differ (*p* ≤ 0.05, two-way ANOVA). White arrows indicate NPC1L1 membrane localization. WT-C, WT mice fed control diet; WT-Z, WT mice fed zeaxanthin diet; db/db-C, db/db mice fed control diet; db/db-Z, db/db mice fed zeaxanthin diet. The original WB images are shown in the Supplementary Materials.

Immunofluorescence imaging revealed distinct genotype- and diet-dependent patterns of NPC1L1 localization along the ileal brush border, with APN used as a marker of membrane protein localization (Figure 2A). In db/db mice fed the control diet, NPC1L1 exhibited a strong membrane-associated distribution that was more pronounced than that observed in WT mice. Zeaxanthin supplementation did not enhance NPC1L1 brush border localization in WT animals. In contrast, in db/db mice, zeaxanthin markedly reduced NPC1L1 membrane localization and promoted intracellular redistribution (indicated by arrows), suggesting altered trafficking or retention dynamics. Despite these differences in subcellular localization, total NPC1L1 protein abundance did not differ significantly between db/db dietary groups but was elevated in db/db mice relative to WT controls, as confirmed by immunoblotting (Figure 2B,C).

### 3.3. Zeaxanthin Modulates Hepatic Lipid Uptake, Oxidation-Related Gene Expression, and Lipogenic Signaling in db/db Mice

**Figure 3:**
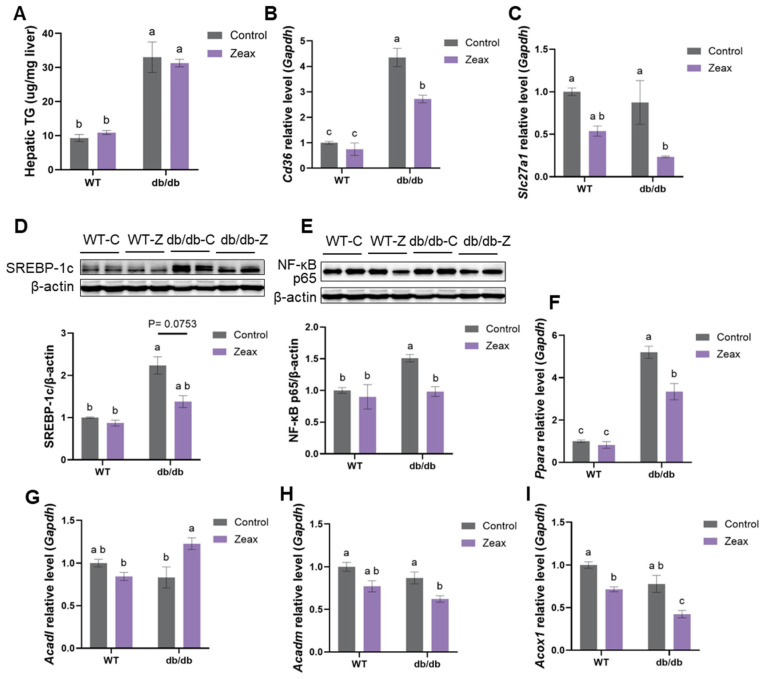
Zeaxanthin attenuates hepatic lipid accumulation and modulates lipid uptake, oxidation, and lipogenic signaling. (**A**) Hepatic triglyceride (TG) concentrations; (**B**,**C**) relative mRNA expression of *Cd36* (**B**) and *Slc27a1* (**C**); (**D**,**E**) representative immunoblots (upper panels) and corresponding quantification (lower panels) of SREBP-1c (**D**) and nuclear factor kappa B (NF-κB p65) (**E**) proteins in liver tissue from wild-type (WT) and db/db mice; and (**F**–**I**) relative mRNA expression of *Ppara* (**F**), *Acadl* (**G**), *Acadm* (**H**), and *Acox1* (**I**) in livers of WT and db/db mice fed a control or zeaxanthin-supplemented diet. Values are presented as mean ± SEM (n = 6/group). Means without a common letter differ (*p* < 0.05, two-way ANOVA). WT, wild type; WT-C, WT mice fed control diet; WT-Z, WT mice fed zeaxanthin diet; db/db-C, db/db mice fed control diet; db/db-Z, db/db mice fed zeaxanthin diet. The original WB images are shown in the Supplementary Materials.

To evaluate the potential effects of zeaxanthin on hepatic lipid metabolism, we quantified liver TG contents and examined transcriptional and protein markers related to lipid uptake, fatty acid β-oxidation, and lipogenesis. db/db mice exhibited significantly elevated hepatic TG contents, which were not reduced by zeaxanthin supplementation (Figure 3A). However, zeaxanthin markedly downregulated the expression of the fatty acid uptake genes *CD36* and *Slc27a1/Fatp1* in db/db mice (Figure 3B,C), suggesting suppression of hepatic fatty acid transport. Hepatic lipogenic and inflammatory signaling were assessed by measuring SREBP-1c and NF-κB p65 protein abundance (Figure 3D–G). db/db mice exhibited elevated hepatic SREBP-1c and NF-κB p65 protein levels, both of which were reduced by zeaxanthin treatment. Although the reduction in SREBP-1c did not reach statistical significance (*p* = 0.075), the findings suggest attenuation of hepatic lipogenic and inflammatory signaling.

We next examined the hepatic expression of fatty acid β-oxidation-related genes (Figure 3F–I). Zeaxanthin reduced peroxisome proliferator-activated receptor α (*Ppara*) expression in db/db mice and produced differential effects among downstream oxidation-related genes (Figure 3F). In diabetic mice, zeaxanthin increased acyl-CoA dehydrogenase long chain (*Acadl*) expression while decreasing acyl-CoA dehydrogenase medium chain (*Acadm*) and acyl-CoA oxidase-1 (*Acox1*) expression (Figure 3G–I). Acox1, a key enzyme involved in peroxisomal β-oxidation of very long-chain fatty acids, was reduced in both genotypes following zeaxanthin treatment. Collectively, these transcriptional changes suggest that zeaxanthin modulates hepatic lipid metabolic gene expression in a pathway-specific manner rather than uniformly enhancing fatty acid β-oxidation.

### 3.4. Zeaxanthin Exerts Limited Effects on Mitochondrial Dynamics in db/db Mice

**Figure 4:**
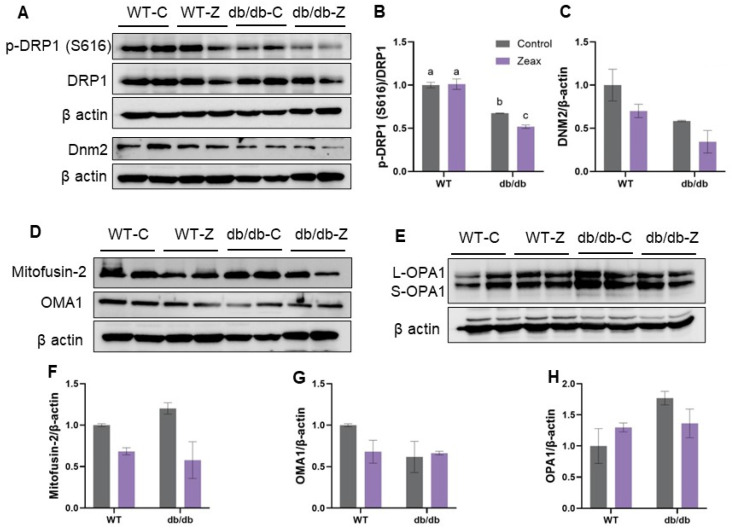
Zeaxanthin exerts limited effects on mitochondrial dynamics in db/db mice. Representative immunoblots and corresponding quantification of phosphorylated dynamin-related protein 1 (p-DRP1) and total DRP1 (**A**,**B**), dynamin 2 (DNM2) (**A**,**C**), mitofusin-2 (MFN2) (**D**,**F**), OMA1 (**D**,**G**), and optic atrophy 1 (OPA1) (**E**,**H**) proteins in liver tissue from wild-type (WT) and db/db mice fed a control or zeaxanthin-supplemented diet. Values are presented as mean ± SEM (n = 6 per group). Means without a common letter differ (*p* < 0.05, two-way ANOVA). WT, wild type; WT-C, WT mice fed control diet; WT-Z, WT mice fed zeaxanthin diet; db/db-C, db/db mice fed control diet; db/db-Z, db/db mice fed zeaxanthin diet. The original WB images are shown in the Supplementary Materials.

Because zeaxanthin is catabolized in mitochondria by BCO2 [36], and BCO2 expression was reduced in db/db mice (Figure 1E), we next examined whether zeaxanthin feeding influences mitochondrial dynamics relevant to β-oxidation. In the liver of db/db mice, zeaxanthin reduced the ratio of phosphorylated DRP1 to total DRP1 (Figure 4A,B). However, DNM2 levels were unchanged, and markers of mitochondrial fusion, including mitofusin-2, OMA1, and OPA1, did not differ among groups (Figure 4A,C–H). These results indicate a modest and selective effect of zeaxanthin on DRP1-associated mitochondrial fission, without broad alterations in mitochondrial dynamics. The functional significance of this change, particularly its contribution to hepatic β-oxidation, remains to be determined.

### 3.5. Zeaxanthin Selectively Modulates Hepatic Glucose Regulatory Pathways in db/db Mice

**Figure 5:**
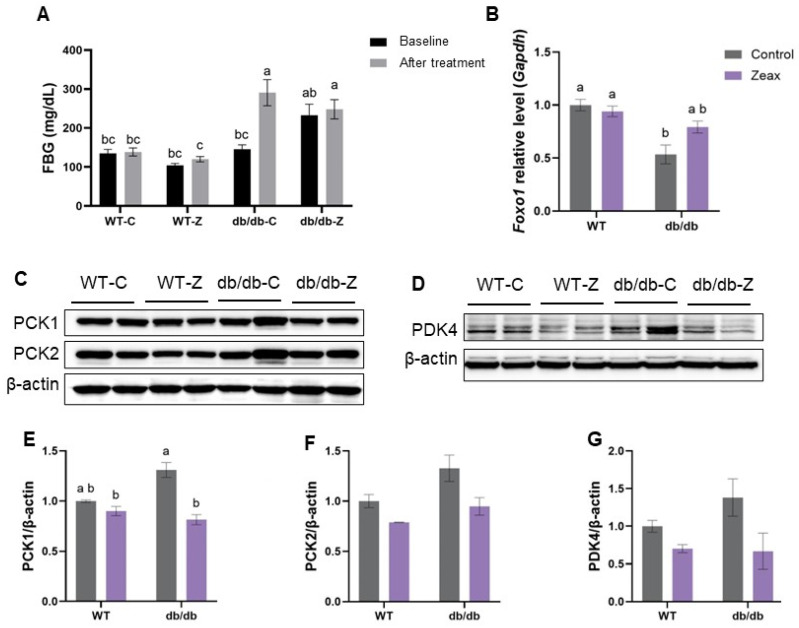
Zeaxanthin selectively modulates hepatic glucose regulatory pathways in db/db mice. (**A**) Fasting blood glucose (FBG) levels measured at the initiation of the dietary intervention (baseline) and at the end of the intervention period (after treatment); (**B**) relative hepatic mRNA expression of *Foxo1*; and (**C**–**G**) representative immunoblots (upper panels) and corresponding quantification (lower panels) of phosphoenolpyruvate carboxykinase 1 (PCK1) (**C**,**D**), phosphoenolpyruvate carboxykinase 2 (PCK2) (**E**,**F**), and pyruvate dehydrogenase kinase 4 (PDK4) (**G**) proteins in liver tissue from wild-type (WT) and db/db mice fed a control or zeaxanthin-supplemented diet. Values are presented as mean ± SEM (n = 6 per group). Means without a common letter differ (*p* < 0.05, two-way ANOVA). WT, wild type; WT-C, WT mice fed control diet; WT-Z, WT mice fed zeaxanthin diet; db/db-C, db/db mice fed control diet; db/db-Z, db/db mice fed zeaxanthin diet. The original WB images are shown in the Supplementary Materials.

To assess glycemic control, fasting blood glucose (FBG) was measured at baseline and again at four weeks of zeaxanthin intervention. At study initiation (4 weeks of age), db/db mice did not exhibit significantly elevated FBG compared to WT controls (Figure 5A). However, substantial variability was observed in the baseline FBG values within the db/db-zeaxanthin (db/db-Z) group, relative to the db/db-control (db/db-C) group. By 8 weeks of age, FBG remained unchanged in WT mice regardless of diet, whereas db/db control mice exhibited marked elevation of FBG levels of approximately 300 mg/dL (Figure 5A). Zeaxanthin supplementation did not significantly lower FBG in db/db mice, suggesting that hyperglycemia during this early stage of disease progression may be relatively insensitive to dietary zeaxanthin under the conditions tested.

To further examine hepatic glucose regulatory pathways, we quantified transcriptional and protein markers of gluconeogenesis, including forkhead box protein O1 (Foxo1), PCK1, PCK2, and PDK4 (Figure 5B–G). Foxo1 gene expression was significantly decreased in diabetic mice and only modestly increased by zeaxanthin, suggesting limited regulatory effects. Among downstream gluconeogenic enzymes, zeaxanthin reduced hepatic PCK1 protein abundance in db/db mice, whereas PCK2 and PDK4 remained unchanged. These findings suggest that zeaxanthin selectively modulates components of hepatic gluconeogenic signaling without significantly improving fasting hyperglycemia under the conditions tested.

### 3.6. Zeaxanthin Modifies Gut Microbiome Diversity and Phylum-Level Composition in db/db Mice

**Figure 6:**
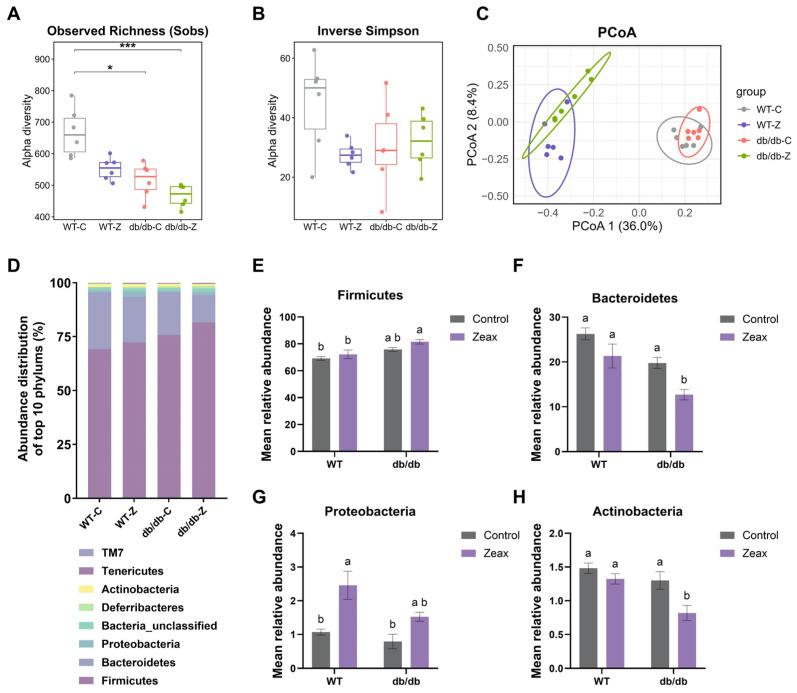
Zeaxanthin modifies cecal microbiome diversity and phylum-level composition in db/db mice. (**A**,**B**) Alpha diversity assessed by observed richness (Sobs; (**A**)) and the inverse Simpson index (**B**). (**C**) Beta diversity visualized by principal coordinate analysis (PCoA) based on Bray–Curtis dissimilarity. (**D**) Stacked bar plots showing microbial composition at the phylum level. (**E**–**H**) Relative abundance of Firmicutes (**E**), Bacteroidetes (**F**), Proteobacteria (**G**), and Actinobacteria (**H**) in wild-type (WT) and db/db mice fed a control or zeaxanthin-supplemented diet. Values are presented as mean ± SEM (n = 6 per group). Statistical significance was determined by two-way ANOVA. Means without a common letter differ (*p* < 0.05). * *p* ≤ 0.05 and *** *p* ≤ 0.001 indicate significant differences in Sobs relative to WT-C for db/db-C and db/db-Z groups, respectively (two-way ANOVA). WT, wild type; WT-C, WT mice fed control diet; WT-Z, WT mice fed zeaxanthin diet; db/db-C, db/db mice fed control diet; db/db-Z, db/db mice fed zeaxanthin diet.

Finally, we determined whether zeaxanthin alters the intestinal microbial community composition and function in cecal samples using 16S rRNA sequencing integrating with PICRUSt2 pathway prediction. The 16S rRNA sequencing depth ranged from 43,763 to 69,624 reads per sample, and all samples were rarefied to 43,763 reads per sample for downstream analyses. Alpha diversity (observed richness, Sobs) of cecal microbiota was significantly reduced in db/db mice compared with WT controls (*p* < 0.05), and this reduction was not reversed by zeaxanthin supplementation (Figure 6A). The inverse Simpson index showed no significant differences among groups (Figure 6B), suggesting that changes in richness were not accompanied by detectable shifts in evenness.

Beta-diversity analysis using PCoA revealed clear clustering separation by diet rather than by genotype (WT vs. db/db), although partial genotypic separation was still evident (Figure 6C). Taxonomic profiling showed that the gut microbiota across all groups was dominated by Firmicutes and Bacteroidetes, with smaller contributions from Proteobacteria, Actinobacteria, Deferribacteres, and Tenericutes (Figure 6D). In WT mice, zeaxanthin supplementation did not markedly alter the relative abundance of Firmicutes, Bacteroidetes, or Actinobacteria, but increased Proteobacteria. In contrast, db/db mice exhibited more pronounced compositional shifts in response to zeaxanthin: the relative abundances of Actinobacteria and Bacteroidetes were significantly reduced compared with db/db mice fed the control diet. Proteobacteria abundance, which was elevated by zeaxanthin in WT mice, was not significantly altered in db/db mice, whose baseline levels were comparable between diets. Firmicutes abundance remained stable in control-fed groups but increased modestly in zeaxanthin-treated db/db mice (Figure 6E–H).

### 3.7. Zeaxanthin Selectively Reshapes Gut Microbial Genera in db/db Mice

**Figure 7:**
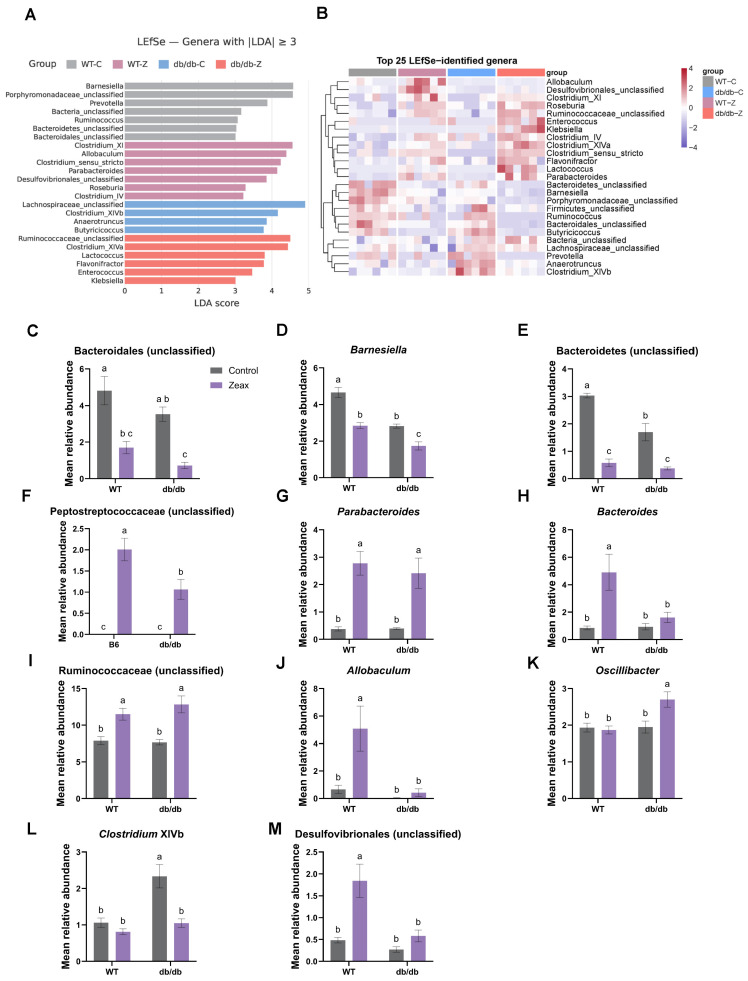
Zeaxanthin selectively reshapes cecal microbial genera in db/db mice. (**A**) Relative abundance of the top 25 bacterial genera across all samples. (**B**–**M**) Relative abundance of selected genera, including *Bacteroidales* (unclassified) (**B**), *Barnesiella* (**C**), *Bacteroidetes* (unclassified) (**D**), *Peptostreptococcaceae* (unclassified) (**E**), *Parabacteroides* (**F**), *Bacteroides* (**G**), *Ruminococcaceae* (unclassified) (**H**), *Allobaculum* (**I**), *Oscillibacter* (**J**), *Clostridium* cluster XIVb (**K**), *Desulfovibrionales* (unclassified) (**L**), and *Desulfovibrionales* (**M**), in wild-type (WT) and db/db mice fed a control or zeaxanthin-supplemented diet. Values are presented as mean ± SEM (n = 6 per group). Statistical significance was determined by two-way ANOVA. Means without a common letter differ (*p* < 0.05). WT, wild type; WT-C, WT mice fed control diet; WT-Z, WT mice fed zeaxanthin diet; db/db-C, db/db mice fed control diet; db/db-Z, db/db mice fed zeaxanthin diet.

To resolve genus-level responses beyond phylum differences, we conducted LEfSe analysis (LDA ≥ 3.0), which revealed distinct microbial discriminants among the treatment groups (Figure 7A). Hierarchical clustering of the top 25 genera further illustrated clear separation between WT and db/db mice, and zeaxanthin supplementation generated a distinct microbial signature within each genotype (Figure 7B).

Several genera associated with mucosal energy harvesting and bile acid metabolism, including unclassified *Bacteroidetes* and *Barnesiella*, were markedly reduced in db/db mice fed the control diet compared with WT mice and remained low or were further suppressed following zeaxanthin supplementation. In contrast, zeaxanthin significantly enriched taxa associated with microbial fermentation and short-chain fatty acid (SCFA) production, including unclassified *Parabacteroides*, *Peptostreptococcaceae*, and *Ruminococcaceae*, in both WT and db/db mice. Zeaxanthin also increased the relative abundance of *Allobaculum*, *Bacteroides*, and unclassified *Desulfovibrionales* specifically in WT mice, whereas it selectively enriched *Oscillibacter* in db/db mice and reduced unclassified *Bacteroidales* in both genotypes. Notably, zeaxanthin reduced the elevated abundance of *Clostridium* XIVb observed in db/db mice, shifting its levels toward those seen in WT controls (Figure 7C–M). This change is notable given prior associations of *Clostridium* cluster XIVb with altered microbial community structure in metabolic disease.

The results indicate that zeaxanthin did not restore overall microbial richness rather promoted selective compositional shifts, enriching fermentation- and SCFA-associated genera while reducing dysbiosis- and inflammation-linked taxa. Such restructuring may contribute to metabolic improvement by altering luminal fermentation, host–microbiome lipid handling, and inflammatory tone.

### 3.8. Zeaxanthin Reshapes Predicted Microbiome Metabolic Pathways in db/db Mice

**Figure 8:**
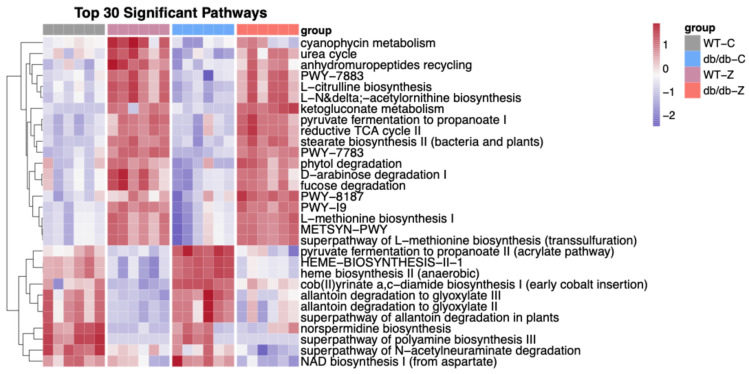
Zeaxanthin reshapes predicted cecal microbiome metabolic pathways in db/db mice. Differentially abundant microbial features and predicted metabolic pathways are associated with zeaxanthin supplementation in wild-type (WT) and db/db mice. LEfSe analysis was used to identify discriminative taxa and pathways, and predicted functional profiling was performed using PICRUSt2 based on 16S rRNA gene sequencing data. Results are presented as relative abundance or linear discriminant analysis (LDA) scores, highlighting pathways related to fermentation, amino acid biosynthesis, and cofactor metabolism across dietary groups. WT, wild type; WT-C, WT mice fed control diet; WT-Z, WT mice fed zeaxanthin diet; db/db-C, db/db mice fed control diet; db/db-Z, db/db mice fed zeaxanthin diet.

To explore whether zeaxanthin-driven taxonomic shifts were accompanied by functional changes, we used PICRUSt2/ggpicrust2 to infer MetaCyc pathways [35] and visualized the top 50 differentially abundant pathways across groups. The results revealed that functional profiles clustered primarily by diet rather than genotype, with relatively modest separation between WT and db/db mice under control conditions (Figure 8). Zeaxanthin supplementation induced coordinated shifts in pathways related to amino acid biosynthesis, fermentation, and cofactor metabolism in both genotypes.

In db/db mice fed the control diet, several inferred MetaCyc pathways were enriched relative to WT controls, including pyruvate fermentation to propanoate II (acrylate pathway), heme biosynthesis II (anaerobic), glycolysis V, cobalamin (vitamin B12) precursor biosynthesis [cob(II)yrinate a,c-diamide biosynthesis], the S-adenosyl-L-methionine (SAM) cycle I, and allantoin degradation to glyoxylate (II/III). Collectively, these enrichments suggest a microbial community with increased predicted capacity for anaerobic carbon fermentation and energy metabolism, along with elevated potential for cofactor production (heme and cobalamin) and one-carbon/methyl-donor metabolism (SAM cycle). The increased allantoin-to-glyoxylate degradation pathways may also reflect altered microbial handling of purine/uric-acid–related metabolites, consistent with a metabolic-stress environment.

In WT mice, zeaxanthin supplementation was associated with enrichment of multiple inferred MetaCyc pathways related to central carbon metabolism, amino acid biosynthesis, and microbial cofactor metabolism. These included pathways involved in TCA cycle activity (oxidative and reductive branches), pyruvate fermentation to propanoate, sugar degradation (D-arabinose and fucose), and ketogluconate metabolism, suggesting an increased predicted capacity for microbial energy metabolism and fermentation. Zeaxanthin also enriched pathways related to methionine and one-carbon metabolism, including the S-adenosyl-L-methionine (SAM) cycle, methionine biosynthesis, and transsulfuration, as well as lysine, threonine, and methionine biosynthesis superpathways. Additional enrichment was observed in pathways associated with nucleotide salvage, tRNA processing, and urea cycle-related metabolism, indicating broader shifts in microbial biosynthetic and nitrogen-handling potential.

When comparing zeaxanthin-treated db/db mice with zeaxanthin-treated WT mice, distinct differences in inferred microbial functional capacity were observed. Several MetaCyc pathways were enriched in db/db mice, including guanosine nucleotide degradation, bacterial anaerobic L-ascorbate degradation, and pathways related to methionine and one-carbon metabolism, such as the S-adenosyl-L-methionine biosynthesis superpathway, L-methionine biosynthesis I (METSYN-PWY), and the methionine biosynthesis/transsulfuration superpathway. Enrichment was also detected in broader amino acid biosynthetic programs, including the superpathway of L-lysine, L-threonine, and L-methionine biosynthesis and the aspartate superpathway. In contrast, zeaxanthin-treated db/db mice exhibited reduced inferred abundance of pathways related to TCA cycle V (2-oxoglutarate:ferredoxin oxidoreductase), tRNA processing, the superpathway of purine nucleotide salvage, 2-oxobutanoate degradation I, and anhydromuropeptides recycling compared with zeaxanthin-treated WT mice.

## 4. Discussion

Although zeaxanthin is best known for its roles in retinal health and antioxidant defense, its metabolic actions in type 2 diabetes remain incompletely understood. In this study, short-term (4 weeks) zeaxanthin supplementation in young db/db mice during early T2DM progression attenuated body weight gain, modulated hepatic lipid metabolism-related signaling, reduced inflammatory markers, and induced selective remodeling of the gut microbiome. Despite limited effects on fasting blood glucose and hepatic triglyceride accumulation, these findings suggest that zeaxanthin influences early metabolic adaptations primarily through modulation of lipid transport-related pathways, inflammatory tone, and host–microbiome interactions during early disease progression.

### 4.1. Integrated Regulation of Intestinal Lipid Uptake and Hepatic Inflammatory and Metabolic Programming During Early T2DM Progression

At the intestinal level, altered subcellular localization (or internalization) of NPC1L1 suggests that zeaxanthin may influence lipid uptake through post-translational or trafficking-based mechanisms, rather than suppression of gene expression as reported in the literature [15,17,18,19], thereby modulating lipid entry without changing total transporter abundance. Such regulation provides a flexible means to limit excess lipid influx under metabolic stress while preserving essential nutrient absorption. This intestinal “gatekeeping” likely contributes to reduced lipid delivery to the liver and downstream metabolic effects.

Hepatic responses to zeaxanthin in db/db mice were primarily characterized by changes in the expression of genes related to fatty acid transport and lipid metabolism rather than measurable reductions in hepatic triglyceride accumulation. Reduced expression of *CD36* and *Slc27a1/Fatp1* suggests suppression of hepatic fatty acid uptake-related pathways [37,38,39], although these transcriptional changes were not sufficient to significantly lower liver triglyceride contents during the intervention period. Zeaxanthin also produced differential regulation of lipid β-oxidation-related genes, including *Ppara*, *Acadl*, *Acadm*, and *Acox1*, indicating selective modulation of hepatic lipid metabolic gene expression rather than coordinated activation of oxidative metabolism [40,41,42,43].

In contrast, reductions in hepatic NF-κB signaling together with lower circulating KC levels suggest that zeaxanthin more consistently attenuated both local and systemic inflammatory stress under diabetic conditions [44]. Given the recognized contribution of chronic low-grade inflammation to early T2DM progression, these anti-inflammatory effects may represent an important component of the metabolic adaptations associated with zeaxanthin supplementation.

Mitochondrial dynamics showed limited responsiveness to zeaxanthin. Although phosphorylation of DRP1 on Ser616 was reduced, no changes were observed in fusion-related markers, indicating the absence of broad mitochondrial remodeling [45,46,47]. These findings suggest that mitochondrial alterations are modest and likely secondary to reduced hepatic lipid burden rather than a primary driver of metabolic improvement.

Beyond lipid metabolism, zeaxanthin supplementation was associated with reduced hepatic PCK1 protein abundance, a key regulator of gluconeogenesis that is often elevated during insulin resistance [48,49]. However, fasting blood glucose levels were not significantly altered in db/db mice, suggesting that the observed changes in hepatic glucose regulatory signaling were insufficient to produce measurable improvements in systemic glycemia during the intervention period. Interpretation of these findings should also consider the substantial biological variability in fasting blood glucose commonly observed in young db/db mice during early diabetes progression [31]. In the present study, considerable inter-individual variation in baseline FBG was observed within the db/db groups prior to dietary intervention, which may have limited the statistical power to detect relatively modest treatment-associated improvements in systemic glucose regulation during the short-term intervention period. Furthermore, fasting blood glucose measurements represent only a single aspect of glycemic control and may not fully capture dynamic alterations in glucose handling or insulin sensitivity. These findings indicate that zeaxanthin exerted selective effects on hepatic metabolic regulatory pathways under early diabetic conditions.

Collectively, these findings suggest that zeaxanthin modulates early metabolic adaptation through effects on intestinal lipid handling, hepatic inflammatory signaling, and selective metabolic regulatory pathways. Although fasting hyperglycemia and hepatic triglyceride accumulation were not significantly improved, zeaxanthin attenuated local and systemic inflammatory stress, supporting its potential as an early dietary intervention during T2DM progression.

### 4.2. Influence of Early-Life Co-Housing and Genetic Context on Microbiome Separation

A notable strength of this study is the use of a littermate-controlled design, in which WT and db/db homozygous male mice were generated from heterozygous dams and co-housed during the early postnatal period prior to dietary intervention. This approach minimizes variability arising from maternal factors, the environment, and early microbial exposure, all of which are known to strongly shape microbiome composition [50,51]. Consistent with this design, separation of gut microbial communities between WT and db/db mice under control diet conditions was modest compared with studies using animals from separate breeders or housing environments.

Importantly, the co-housing period encompassed the first 3.5–4 weeks of life (accounting for a 3–5 day genotyping turnaround by Transnetyx), a critical window for microbiome establishment, during which db/db mice are not yet severely hyperglycemic and exhibit relatively limited metabolic divergence from WT littermates. These findings suggest that leptin receptor deficiency alone may not strongly determine gut microbiome composition during early life and that maternal and environmental influences likely predominate at this stage.

The relatively limited microbiome divergence observed after four weeks of dietary intervention further supports the concept that early metabolic dysfunction may precede major microbiome restructuring in young db/db mice [50,52]. This controlled design therefore provided an opportunity to evaluate zeaxanthin-associated microbial and metabolic adaptations within a relatively uniform microbial background prior to the onset of advanced diabetes-associated dysbiosis.

### 4.3. Zeaxanthin-Driven Microbiome Restructuring and Predicted Functional Consequences During Early Metabolic Dysfunction

Within the conserved early-life microbial background described above, microbiome responses are best characterized as selective recalibration rather than overt dysbiosis [53,54]. The modest separation between WT and db/db mice under control conditions, together with diet-driven shifts induced by zeaxanthin, indicate that early metabolic dysfunction does not require large-scale restructuring of the gut microbial community [55]. Instead, the microbiome at this stage appears highly plastic and responsive to dietary cues, providing a window in which subtle compositional changes may have disproportionate metabolic consequences. Zeaxanthin-associated changes suggest a shift away from configurations linked to inflammatory stress and toward microbial profiles enriched in fermentation- and short-chain fatty acid-associated taxa. Such restructuring is consistent with a community that supports barrier integrity and limits pro-inflammatory signaling rather than one that promotes excessive energy harvest or metabolic endotoxemia [56,57]. That aligns with reductions in systemic inflammatory markers and improved hepatic metabolic tone in db/db mice, despite minimal changes in fasting glycemia [21].

Predicted functional profiling further suggests that host metabolic status constrains microbial adaptation. Rather than inducing uniform changes across genotypes, zeaxanthin elicited context-dependent shifts in inferred microbial metabolic capacity [58,59]. In WT mice, responses reflected broader biosynthetic and metabolic flexibility, whereas in db/db mice, changes were more selective and consistent with a metabolically stressed environment. This divergence underscores the dynamic interaction between host physiology and microbial function, even when overall community structure remains relatively stable [60,61,62].

Further, these changes should be interpreted cautiously, as they reflect predicted metabolic capacity rather than direct measurements of microbial activity [63]. Within this framework, the microbiome may act as a modulatory interface, translating dietary carotenoid exposure into signals that influence host lipid handling and inflammatory tone during early disease progression, different from the high-fat-induced insulin-resistant mouse models fed zeaxanthin [59]. The concordance between predicted microbial functions and host metabolic responses supports the plausibility of such crosstalk, while highlighting the need for future studies incorporating metagenomics and metabolomics to establish causal relationships.

Together, these findings suggest that zeaxanthin does not reverse advanced microbiome dysbiosis but instead induces modest, selective shifts in microbial composition and functional potential at a stage when host–microbiome plasticity is retained. Although the magnitude of change is limited, the directionality is consistent with improvements in lipid metabolism and inflammatory tone, supporting a contributory, rather than causal role for the microbiome in early metabolic adaptation.

### 4.4. Relevance to Human Nutrition, Dosage Considerations, and Metabolic Context

Zeaxanthin intake in humans is generally low, and circulating levels are further reduced in obesity and insulin resistance [64,65], suggesting that carotenoid insufficiency may accompany early metabolic dysfunction. In this study, a dietary zeaxanthin concentration of 0.02% (*w*/*w*) was used to ensure sufficient exposure during the 4-week intervention [21]. This corresponds to approximately 0.6 mg/day per mouse (~24 mg/kg/day) and a human-equivalent dose of ~2 mg/kg/day (≈140 mg/day for a 70 kg adult). Although substantially higher than typical dietary intake, such dosing is commonly used in rodent studies and should not be directly translated to human consumption levels [5,21,59,66].

A context-dependent observation in this study was the differential effect of zeaxanthin on circulating insulin levels between genotypes. Zeaxanthin modestly increased insulin levels in WT mice, whereas insulin levels were reduced in db/db mice, which exhibited marked hyperinsulinemia under control diet conditions. Importantly, the insulin increase observed in WT mice occurred without accompanying hyperglycemia, body weight gain, hepatic triglyceride accumulation, or increased inflammatory markers, suggesting that this response may not reflect overt insulin resistance under the conditions tested. The mechanisms underlying these genotype-dependent insulin responses remain unclear and were not directly investigated in the current study. One possibility is that differences in intestinal lipid handling, carotenoid absorption, or metabolic state between WT and db/db mice may influence systemic exposure and endocrine responses to zeaxanthin [67,68]. Additional studies will be warranted to investigate the mechanisms underlying these genotype-dependent insulin responses and to determine whether they reflect adaptive metabolic regulation, altered nutrient sensing, or compensatory endocrine responses during early metabolic dysfunction.

The db/db model reflects leptin receptor deficiency, in which metabolic dysfunction is genetically driven rather than primarily diet-induced [59,69]. Accordingly, the limited effects of zeaxanthin on fasting glucose and hepatic triglyceride accumulation do not preclude biologically relevant modulation of adiposity, inflammatory signaling, and metabolic regulatory pathways. Collectively, these findings suggest that zeaxanthin may exert context-dependent effects on early metabolic dysfunction primarily through modulation of lipid handling and inflammatory stress rather than acting as a direct glucose-lowering intervention as reported in the literature [59].

### 4.5. Study Limitations and Future Directions

Several limitations should be considered when interpreting the present findings. First, this study utilized young db/db mice, a genetic model of early-onset T2DM characterized by leptin receptor deficiency, rapid disease progression, hyperphagia, and severe obesity. Although this model is appropriate for investigating genetically driven early metabolic dysfunction, the findings may not fully translate to diet-induced obesity and insulin resistance models [21] or later-stage diabetic disease. In addition, because the intervention was initiated during early disease progression, the observed effects likely reflect modulation of early metabolic and inflammatory adaptation rather than reversal of established diabetic pathology. Future studies using dietary, aging, and later-stage diabetic models will be important to determine the broader applicability of zeaxanthin supplementation across different metabolic contexts.

Second, the intervention duration was relatively short. Zeaxanthin supplementation was administered for 4 weeks during early disease progression, allowing assessment of early metabolic, inflammatory, and molecular responses. However, this design does not determine whether the observed effects are sustained during prolonged treatment or whether zeaxanthin can influence progression toward advanced diabetic complications, including severe insulin resistance, hepatic steatosis, or pancreatic β-cell dysfunction. Longer-term studies will therefore be necessary to evaluate the durability and physiological significance of the observed responses.

Third, although fasting blood glucose and circulating insulin levels were measured, direct assessment of systemic insulin sensitivity was not performed. Glucose tolerance tests, insulin tolerance tests, hyperinsulinemic-euglycemic clamp studies, and β-cell functional analyses would be necessary to determine whether zeaxanthin influences insulin sensitivity, glucose utilization, or endocrine pancreatic function. This limitation is particularly relevant given the genotype-dependent insulin responses observed in WT and db/db mice following zeaxanthin supplementation. Additional studies will be warranted to investigate the mechanisms underlying these differential insulin responses and to determine whether they reflect adaptive metabolic regulation, altered nutrient sensing, compensatory endocrine responses, or differences in carotenoid bioavailability between genotypes.

Fourth, hepatic lipid metabolism was evaluated primarily through transcriptional and protein markers rather than direct measurements of lipid flux, fatty acid oxidation, or mitochondrial respiration. Although zeaxanthin modulated several lipid metabolism-related pathways, these molecular changes were not accompanied by significant reductions in hepatic triglyceride accumulation. Accordingly, the functional consequences of the observed changes in lipid transport and oxidation-related markers remain to be determined. Similarly, mitochondrial alterations were relatively modest and should be interpreted cautiously in the absence of direct bioenergetic measurements.

Fifth, internal zeaxanthin concentrations in plasma and tissues were not measured. Although dietary exposure was controlled, the absence of tissue-level quantification limits interpretation of exposure–response relationships and prevents direct assessment of zeaxanthin absorption, distribution, and bioavailability under diabetic conditions. This is particularly important given the altered intestinal NPC1L1 localization observed in db/db mice, which may influence carotenoid handling and systemic exposure. Future studies integrating carotenoid quantification with tracer-based metabolic approaches will be important to define the relationship between zeaxanthin exposure and metabolic outcomes.

Sixth, microbiome analyses relied on 16S rRNA sequencing and predictive functional profiling rather than direct metabolite measurements or mechanistic validation. Although these approaches provided insight into microbial community structure and predicted metabolic function, they cannot establish causal relationships between microbiome alterations and host metabolic responses. Furthermore, microbiome depletion, fecal microbiota transplantation, and gnotobiotic validation studies were not performed. Future integration of metabolomics, microbial transfer approaches, and mechanistic microbiome studies will be necessary to identify microbiome-derived mediators associated with zeaxanthin supplementation and to establish causality within the host–microbiome interaction.

Finally, only male mice were included in this study, and group sizes were modest (n = 6 per group) and derived from a limited number of litters and cages. Although sufficient to detect several metabolic and molecular endpoints, this design may limit assessment of sex-specific responses, inter-individual variation, and cage-level microbiome variability. Future studies including female mice, larger cohorts, and expanded cage randomization will strengthen reproducibility and generalizability.

In addition, although FD&C color additives were included solely for diet identification as insoluble aluminum lake dye preparations, potential minor effects on host physiology or microbiome composition cannot be completely excluded.

Despite these limitations, the present study provides integrated insight into how dietary zeaxanthin modulates early metabolic and inflammatory responses during T2DM progression. Rather than functioning primarily as a direct glucose-lowering intervention, zeaxanthin appears to influence early-stage metabolic adaptation through coordinated effects on intestinal lipid handling, inflammatory signaling, and host–microbiome interactions, supporting its potential as a nutrition-based strategy for early metabolic intervention.

## 5. Conclusions

This study demonstrates that dietary zeaxanthin modulates early metabolic and inflammatory responses in young db/db mice during early T2DM progression. Using a littermate-controlled design, we show that zeaxanthin influences intestinal lipid handling, hepatic inflammatory and metabolic signaling, and gut microbiome composition and predicted function. Although zeaxanthin did not significantly improve fasting hyperglycemia or hepatic triglyceride accumulation, it altered lipid transport-related and glucose regulatory pathways while attenuating local and systemic inflammatory stress. These findings support the potential of zeaxanthin as a dietary factor that may contribute to early metabolic adaptation and provide a foundation for future studies in diet-induced metabolic models and human populations.

## Figures and Tables

**Table 1:** Diet composition.

Ingredient	Control Diet	Zeaxanthin Diet (0.02%)
	gm	gm
Zeaxanthin (5%, in triglycerides)	0	4.3
Casein	200	200
L-Cystine	3	3
Corn Starch	390.5	390.5
Maltodextrin 10	110	110
Dextrose	150	150
Cellulose, BW200	100	100
Soybean Oil	70	65.7
Mineral Mix S10026	10	10
DiCalcium Phosphate	13	13
Calcium Carbonate	5.5	5.5
Potassium Citrate, 1 H_2_O	16.5	16.5
Vitamin Mix V10001	10	10
Choline Bitartrate	2	2
FD&C Yellow Dye #5	0.025	0
FD&C Red Dye #40	0.025	0
FD&C Blue Dye #1	0	0.05
Total	1080.550	1076.55

The diet composition was provided, and pellet diets were produced by Research Diets, Inc. (New Brunswick, NJ, USA). FD&C color additives were included solely for diet identification as insoluble aluminum lake dye preparations.

**Table 2:** Primer sequence for real-time PCR.

Gene	Forward	Reverse
*CD36*	5′-gga act gtg ggc tca ttg c-3′	5′-cat gag aat gcc tcc aaa cac-3′
*FATP1*	5′-ccg tat cct cac gca tgt gt-3′	5′-ctc cat cgt gtc ctc att gac-3′
*PPARα*	5′-cgt acg gca atg gct tta tc-3′	5′-aac ggc ttc ctc agg ttc tt-3′
*LCAD*	5′-tca atg gaa gca agg tgt tca-3′	5′-gcc acg acg atc acg aga t-3′
*MCAD*	5′-gat gcc atc acc ctc gtg taa c-3′	5′-aag ccc ttt tcc cct gaa g-3′
*ACOX1*	5′-aag aac tcc aga taa ttg gca cct a-3′	5′-ttt cca agc ctc gaa gat gag-3′
*FOXO1*	5′-tca tgg atg gag ata cct tgg a-3′	5′-ctt gac act gtg tgg gaa gct t-3′
*GAPDH*	5′-caa ggt cat cca tga caa ctt tg-3′	5′-ggc cat cca cag tct tct gg-3′

## Data Availability

Data described in the manuscript, code book, and analytic code will be made available upon request.

## Supplementary Materials

The following supporting information can be downloaded at https://www.mdpi.com/article/10.3390/biom16060818/s1, Supplementary Figures show the original images of Figure 2, Figure 3, Figure 4 and Figure 5.

## Abbreviations

ACADL, Acyl-CoA Dehydrogenase Long Chain (LCAD); ACADM, Acyl-CoA Dehydrogenase Medium Chain (MCAD); Acox1, Peroxisomal Acyl-Coenzyme A Oxidase 1; APN, Aminopeptidase N (AKA CD13); BCO2, β-Carotene Oxygenase 2; C, Control (AIN93M) Diet; CD36, Cluster of Differentiation 36; DNM2, Dynamin-2; p-DRP1, Phospho-Dynamin-Related Protein 1 (Ser616); eWAT, Epididymal White Adipose Tissue; FATP1, Long-Chain Fatty Acid Transport Protein 1 (SLC27A1); FBG, Fasting Blood Glucose; FOXO1, Forkhead Box O1; GAPDH, Glyceraldehyde-3-Phosphate Dehydrogenase; KC, Keratinocyte-Derived Chemokine (CXCL1); LCFA, Long-Chain Fatty Acids; MCFA, Medium-Chain Fatty Acids; MFN2, Mitofusin-2; NF-κB, Nuclear Factor Kappa-Light-Chain-Enhancer of Activated B Cells; NPC1L1, Niemann-Pick C1-Like 1; OMA1, Overlapping with the m-AAA Protease 1 Homolog, or Mitochondrial Metallopeptidase; OPA1, Optic Atrophy Protein 1; PCK1, Phosphoenolpyruvate Carboxykinase 1; PCK2, Phosphoenolpyruvate Carboxykinase 2; PCoA, Principal Coordinate Analysis; PDK4, Pyruvate Dehydrogenase Kinase 4; PPARα, Peroxisome Proliferator-Activated Receptor Alpha; SCARB1, Scavenger Receptor Class B Type 1; SCFA, Short-Chain Fatty Acids; SLC27A1, Solute Carrier Family 27 Member 1 (FATP1); SREBP-1c, Sterol Regulatory Element-Binding Protein 1c; T2DM, Type 2 Diabetes Mellitus; TM7, Saccharibacteria; WT, Wild Type; Z, Zeaxanthin diet (AIN93M with 0.02% zeaxanthin); Zeax, Zeaxanthin or Zeaxanthin Diet.
