# Supplementary material for: Zeaxanthin Modulates Early Metabolic and Inflammatory Responses in db/db Mice: Associations with Intestinal Lipid Handling and Gut Microbiome Remodeling
Source: Biomolecules. 2026 Jun 1;16(6):818. doi: 10.3390/biom16060818 (PMC13296385; doi:10.3390/biom16060818)
Supplement: Supplementary file 1 [file biomolecules-16-00818-s001.zip › biomolecules-4259995-supplementary.pdf]

Zeaxanthin Improves Early Metabolic Dysfunction in db/db Mice: Associations with Gut–Liver Metabolism and Microbiome Remodeling

Yashu Tang [1,†], Peiran Lu [1,†], Huimin Chen [1], Siauyen Wong [1], MD Salahuddin [1], Md Mehedi Hasan [1], Sanmi E Alake [1], Yoo Kim [1], McKale Montgomery [2], Winyoo Chowanadisai [1], Brenda J Smith [3], Stephen L Clarke [1], Edralin A Lucas [1], Chwan-Li Shen [4], Minghua Tang [5] and Dingbo Lin [1,*]

Supplemental WB figures

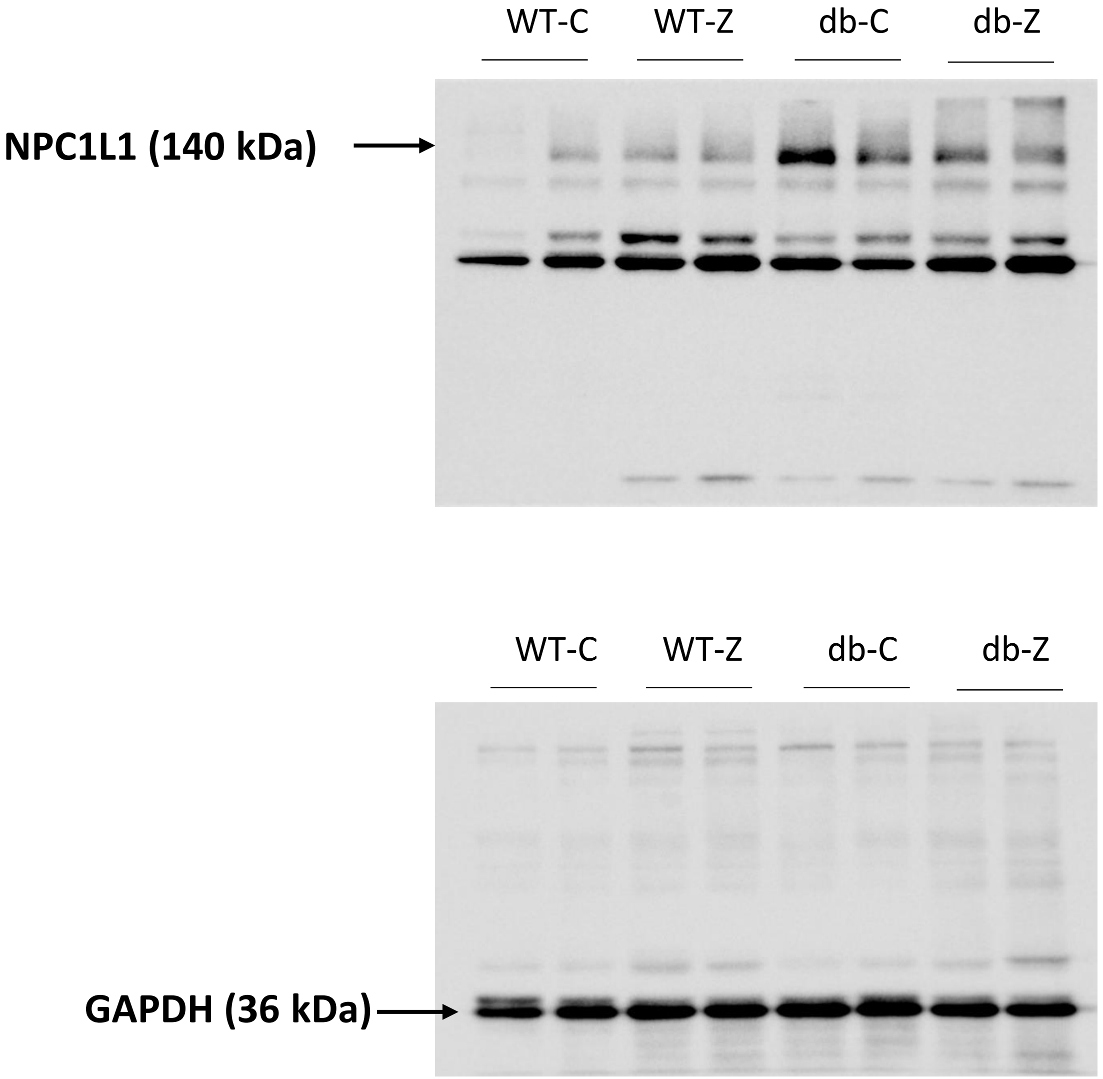
WT-C
WT-Z
db-C
db-Z
NPC1L1 (140 kDa)
WT-C
WT-Z
db-C
db-Z
GAPDH (36 kDa)

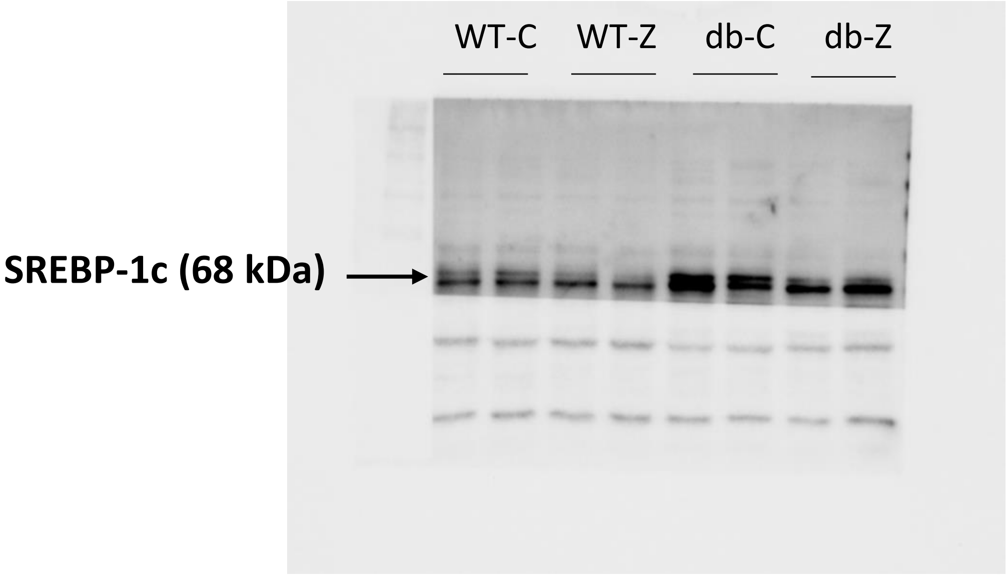
WT-C
WT-Z
db-C
db-Z
SREBP-1c (68 kDa)

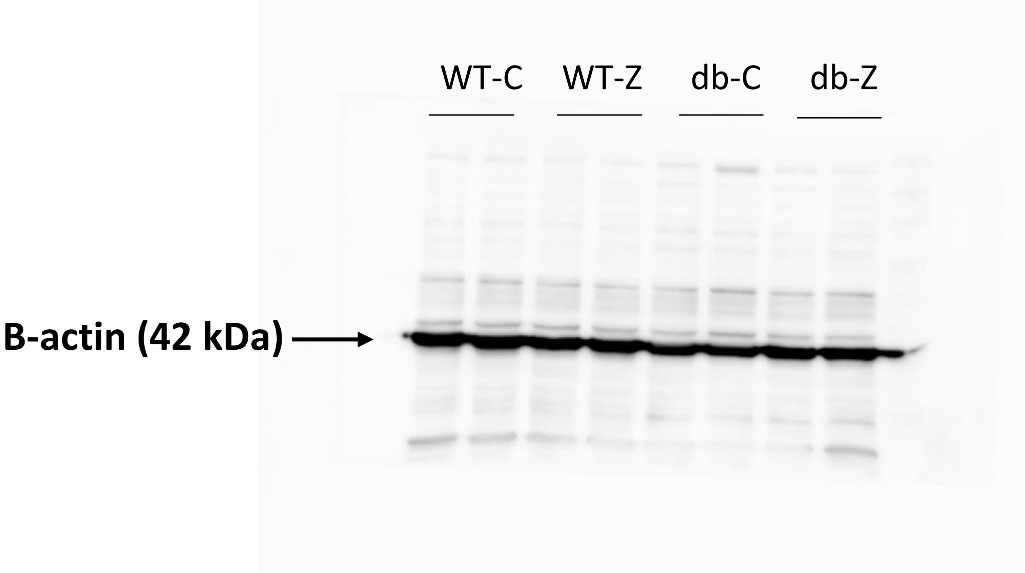
WT-C
WT-Z
db-C
db-Z
B-actin (42 kDa)

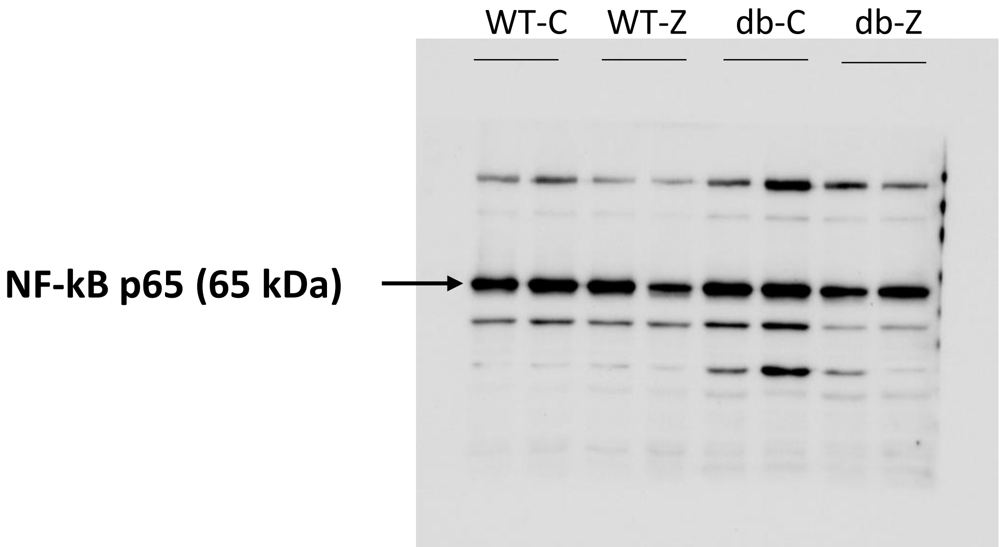
WT-C
WT-Z
db-C
db-Z
NF-kB p65 (65 kDa)

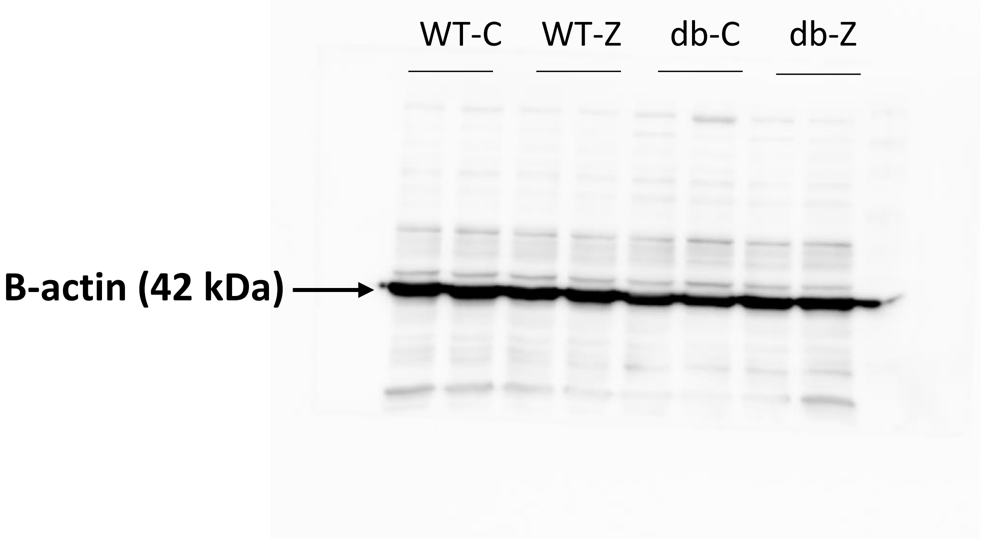
WT-C
WT-Z
db-C
db-Z
B-actin (42 kDa)

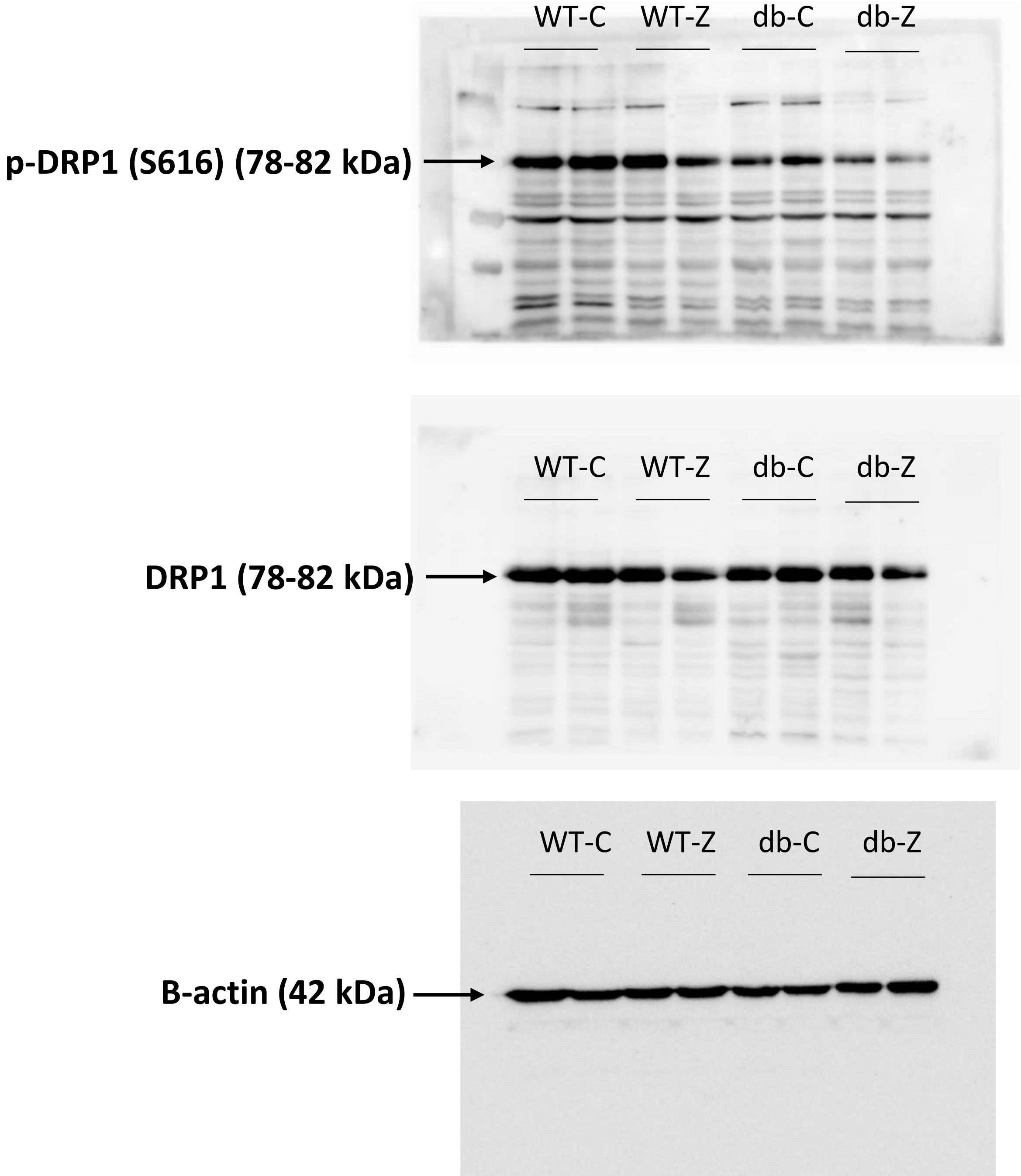
WT-C
WT-Z
db-C
db-Z
p-DRP1 (S616) (78-82 kDa)
WT-C
WT-Z
db-C
db-Z
DRP1 (78-82 kDa)
WT-C
WT-Z
db-C
db-Z
B-actin (42 kDa)

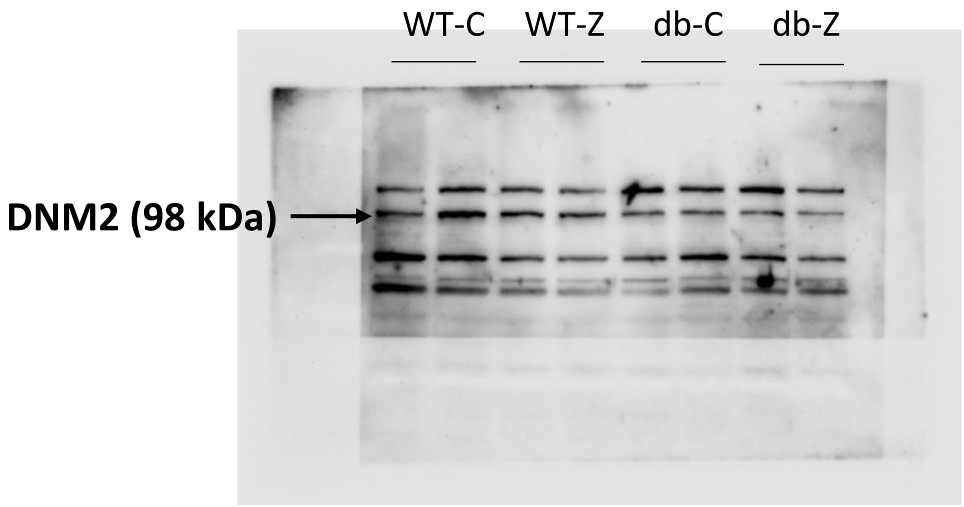
WT-C
WT-Z
db-C
db-Z
DNM2 (98 kDa)

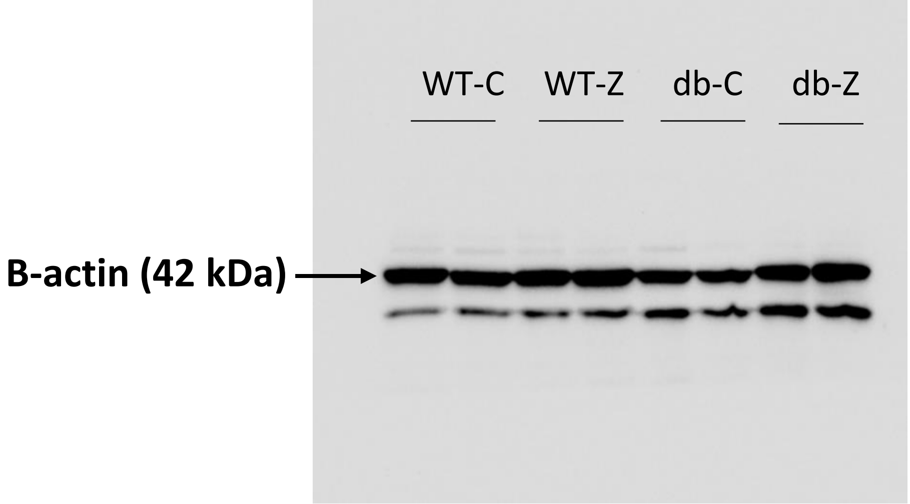
WT-C
WT-Z
db-C
db-Z
B-actin (42 kDa)

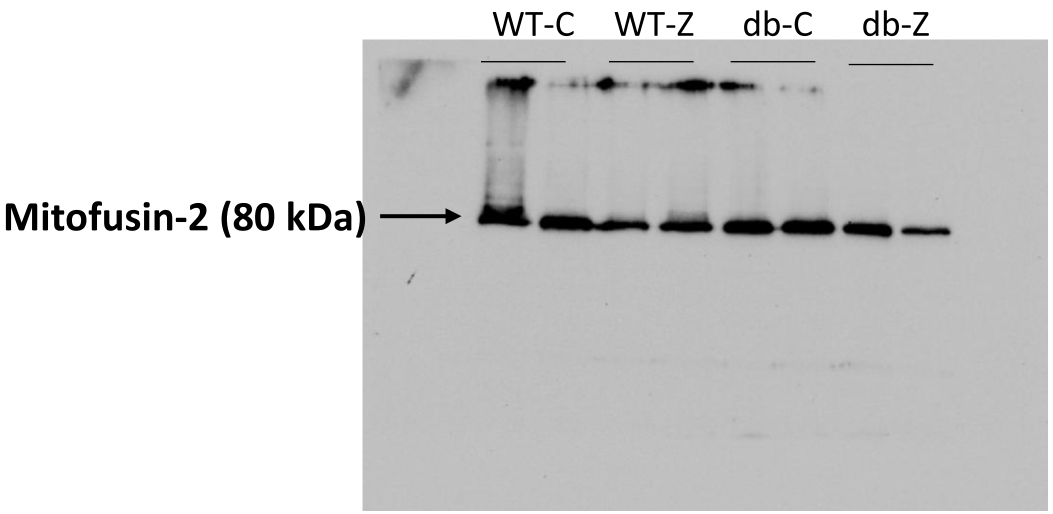
WT-C
WT-Z
db-C
db-Z
Mitofusin-2 (80 kDa)

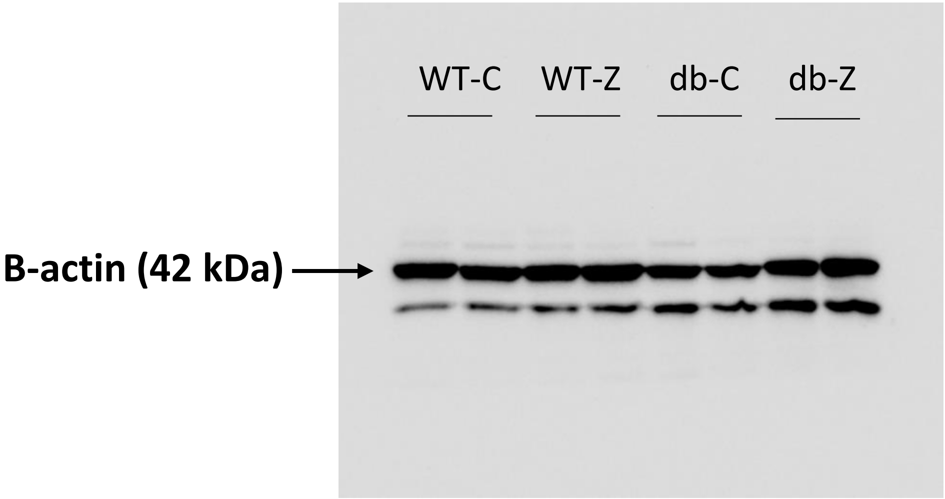
WT-C
WT-Z
db-C
db-Z
B-actin (42 kDa)

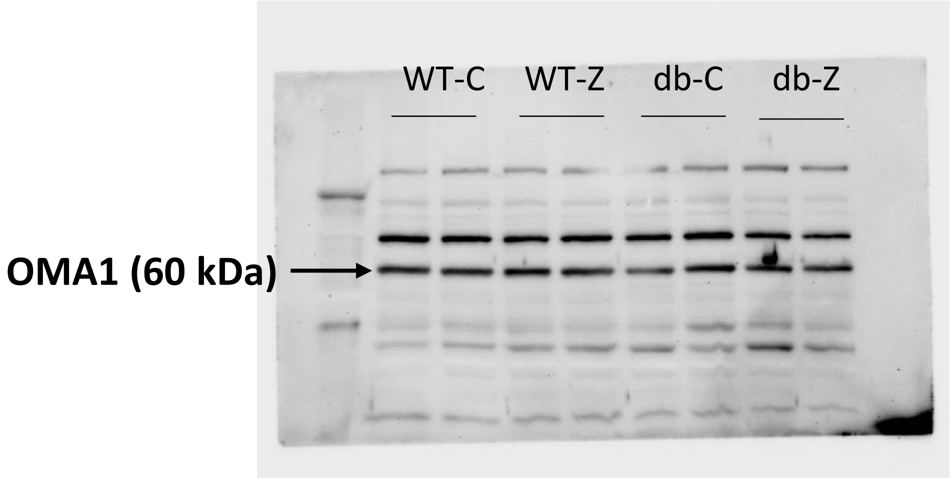
WT-C
WT-Z
db-C
db-Z
OMA1 (60 kDa)

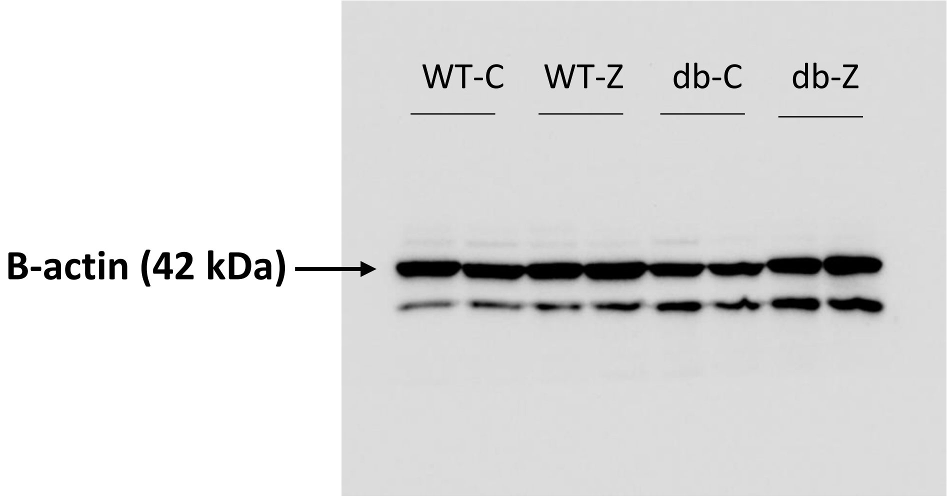
WT-C
WT-Z
db-C
db-Z
B-actin (42 kDa)

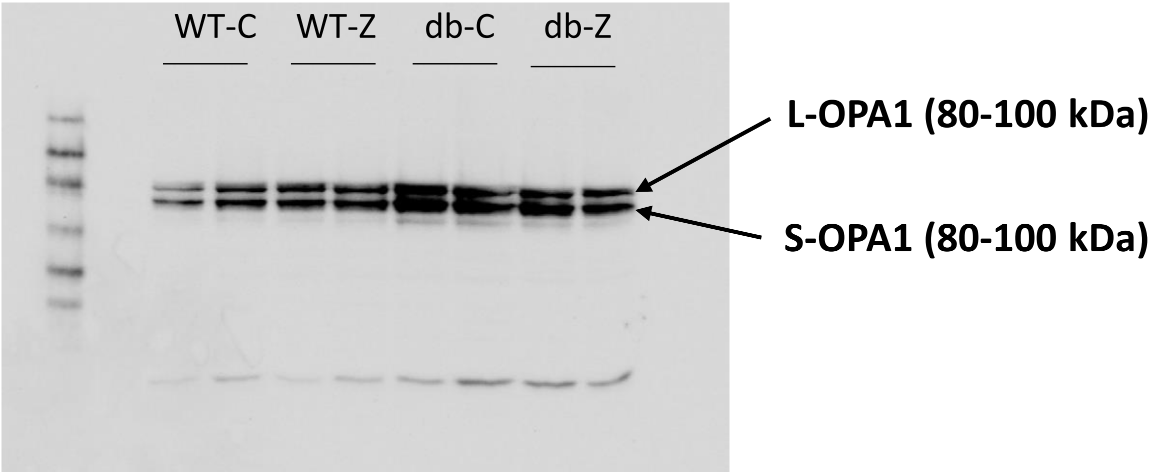
WT-C
WT-Z
db-C
db-Z
L-OPA1 (80-100 kDa)
S-OPA1 (80-100 kDa)

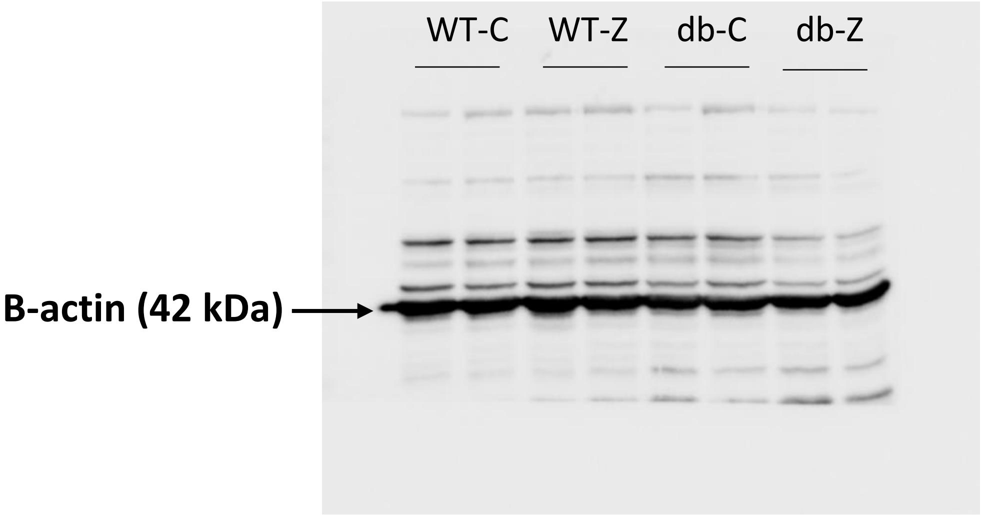
WT-C
WT-Z
db-C
db-Z
B-actin (42 kDa)

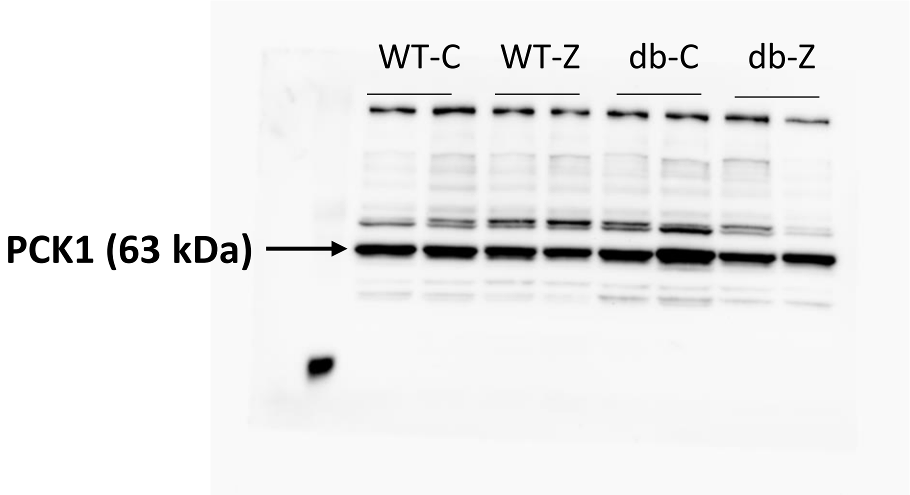
WT-C
WT-Z
db-C
db-Z
PCK1 (63 kDa)

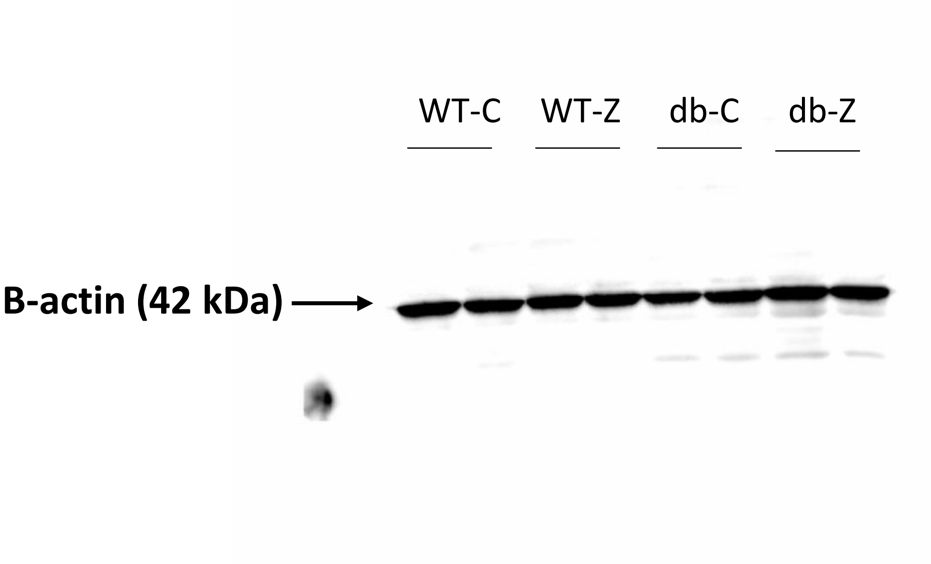
WT-C
WT-Z
db-C
db-Z
B-actin (42 kDa)

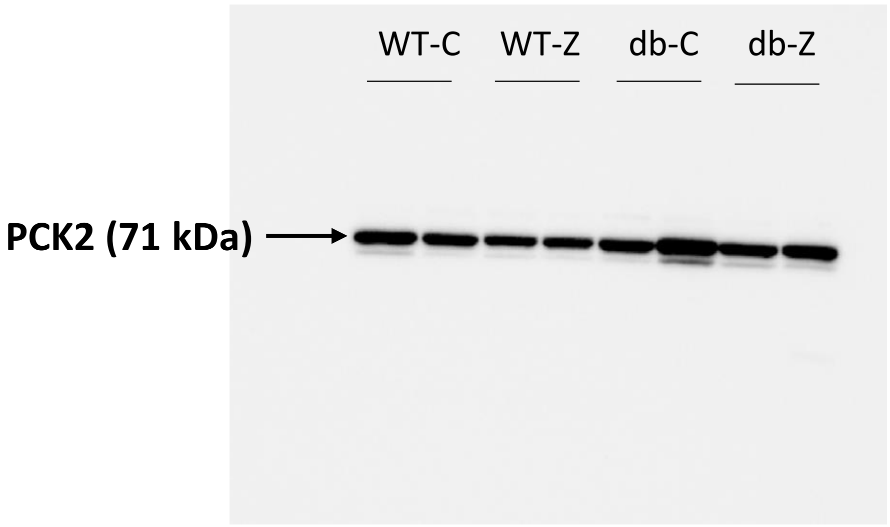
WT-C
WT-Z
db-C
db-Z
PCK2 (71 kDa)

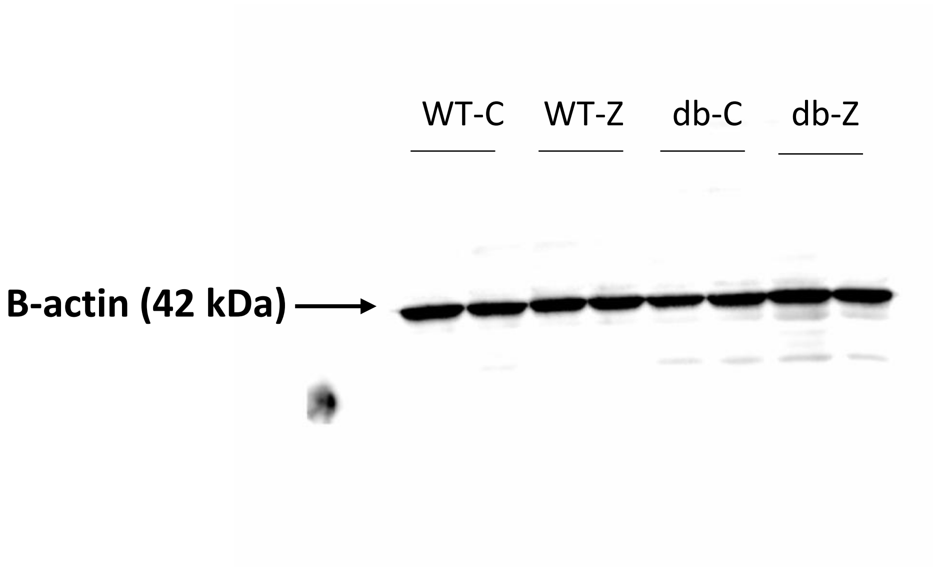
WT-C
WT-Z
db-C
db-Z
B-actin (42 kDa)

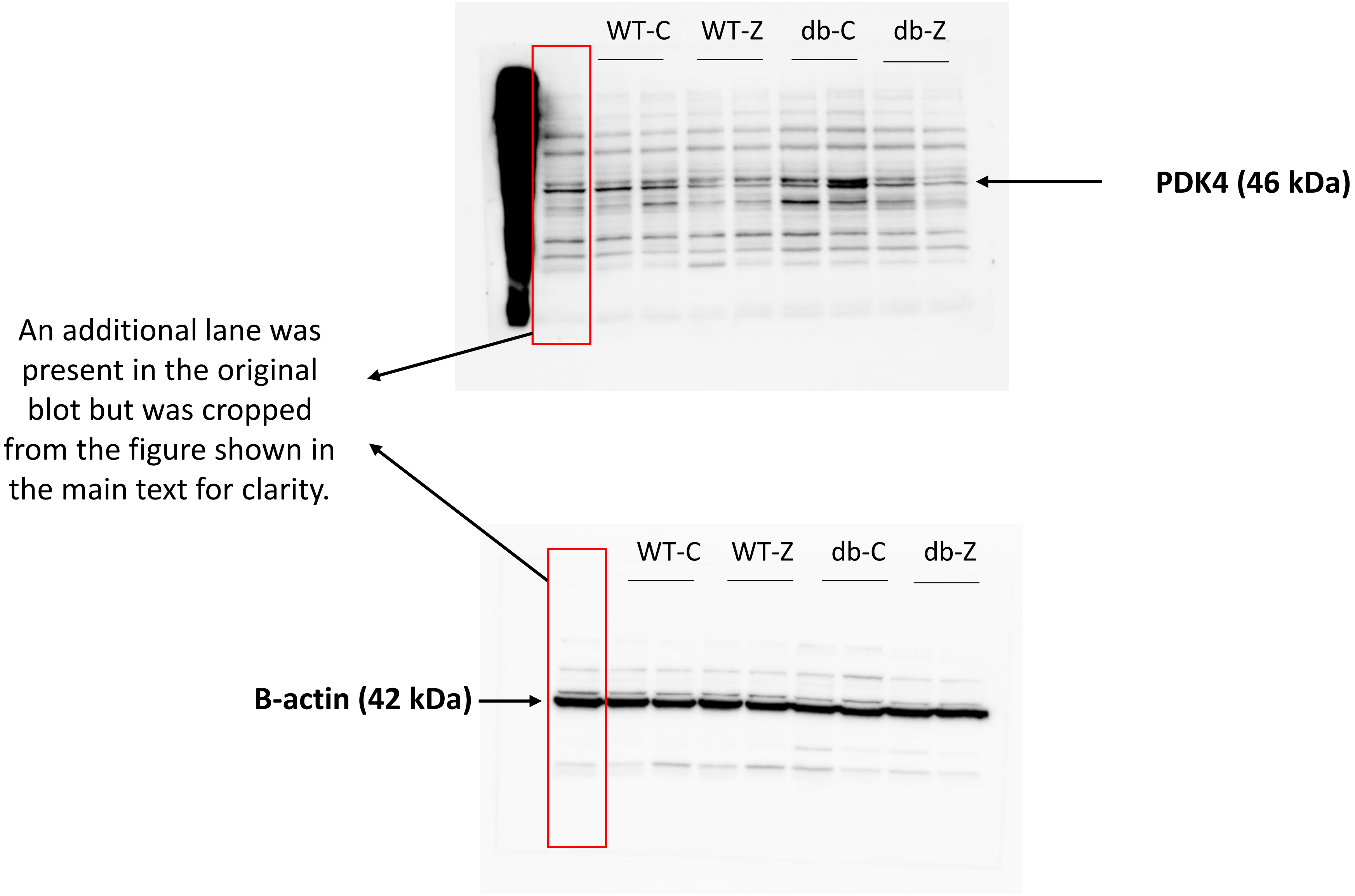
WT-C
WT-Z
db-C
db-Z
PDK4 (46 kDa)
An additional lane was present in the original blot but was cropped from the figure shown in the main text for clarity.
WT-C
WT-Z
db-C
db-Z
B-actin (42 kDa)
